# Ultrastructural investigation and *in vitro* recapitulation of spermatid differentiation in a potential bio-indicator species – The marine invertebrate *Galeolaria gemineoa* (Polychaeta: Serpulidae)

**DOI:** 10.1371/journal.pone.0183986

**Published:** 2017-08-29

**Authors:** Yonggang Lu, Robert John Aitken, Minjie Lin

**Affiliations:** Priority Research Centre for Reproductive Science, School of Environmental and Life Sciences, Faculty of Science, University of Newcastle, Callaghan, New South Wales, Australia; Universite Clermont Auvergne, FRANCE

## Abstract

*Galeolaria gemineoa* is a sessile broadcast-spawning marine invertebrate, whose spermatozoa have been regarded as a sensitive indicator for water quality monitoring. In this study, 10 steps of spermiogenesis have been identified at the ultrastructural level and this differentiation process has been recapitulated *in vitro* up to the point of spermiogenesis (step 7–9 spermatids). On completion of the second meiosis, newly formed spermatids were detached from the seminiferous epithelium and released to the lumen of each germinal chamber. These spermatids were present in pairs and interconnected by a cytoplasmic bridge throughout the entire spermiogenic process. On the basis of morphological events such as formation of the acrosome, elongation of the flagellum, and condensation of the nucleus, spermiogenesis has been temporally divided into Golgi phase, acrosomal phase and maturation phase. During the Golgi phase, proacrosomal vesicles appeared at the posterior pole of the spermatids and gradually fused into a proacrosomal vacuole. Simultaneously, the distal centriole docked onto the plasma membrane and gave rise to a formative flagellum. The acrosomal phase was characterised by differentiation of the acrosome, condensation of the chromatin and formation of a mitochondrial sheath surrounding the initial portion of the flagellum. During the maturation phase, the fully differentiated acrosome migrated to the anterior pole and excess cytoplasm was extruded from the spermatids in the form of residual bodies. In addition, we successfully induced step 1–3 spermatids to differentiate into the step 7–9 spermatids in both male germinal fluid and 10% foetal bovine serum in RPMI 1640 medium, but failed to replicate this process in female or boiled male germinal fluids. This finding supports our concept that spermatid differentiation in this species is dependent on intrinsic developmental programming and does not require input from accompanying nurse cells.

## Introduction

Polychaetes are segmented invertebrates predominantly occupying all marine habitats, ranging from the supralittoral zone to abyssal sediments [1]. They form the largest taxon in Annelida with more than 13,000 species in 83 families [2]. *Galeolaria gemineoa* is an Australian native marine polychaete that ubiquitously inhabits the intertidal region of the south-eastern coast, with a broad geographical range over 4,500 km [3, 4]. A number of the life history characteristics of this species endow it with great potential as both an experimental model for toxicity testing and a bio-indicator species for coastal water quality monitoring.

As a serpulid, *G*. *gemineoa* builds calcareous tubes that form 5 to 15 cm thick assemblages on hard surfaces such as rock revetments, with an extremely high density of up to 10 individuals per cm^3^ [5]. *G*. *gemineoa*’s body size ranges from 10 to 30 mm in length and 0.8 to 2.7 mm in thoracic width [5]. This annelid may be a valuable bio-indicator species because it easy to collect as a consequence of being intertidal and gregarious, and is extremely sensitive to marine pollutants [6]. Furthermore, as a suspension-feeding species, *G*. *gemineoa* maximises its exposure to suspended hazardous substances in the ambient water as they process large volume of water during foraging activities [7]. Waring et al. [8] demonstrated that this species had a higher capacity to accumulate toxic heavy metals including mercury, silver and zinc compared with several other marine polychaetes tested (*Australonereis ehlersi*, *Australonuphis parateres*, *Lumbrinereis* spp., *Marphysa* spp., *Notomastus estuaries*, *Scoloplos simplex*, and *Sigalion* spp.). In reproductive terms, adult male and female *G*. *gemineoa* are filled with viable free-floating gametes in their abdomens throughout the year [9, 10]. As a typical broadcast-spawning species, *G*. *gemineoa* sheds its gametes directly into the ambient water, where fertilisation subsequently takes place [11, 12]. As a result, the gametes of this species are chronically exposed to toxic substances in the local environment before, during and after insemination.

Recent studies have also demonstrated that the behaviour of *G*. *gemineoa* spermatozoa can be used as a feasible and sensitive indicator for the detection of changes in coastal water quality. Specifically, Falkenberg et al. [13] discovered that motile spermatozoa of *G*. *gemineoa* were able to adhere to the bottom of tissue culture well-plates while immotile cells could not. The percentage of spermatozoa accumulated against the well-plate surface was found to have a strong positive relationship with fertilisation rates. Therefore, a bioassay system, Sperm Accumulated Against Surface, was developed as a rapid and reliable tool for biomonitoring purposes [13]. Schlegel et al. [14] indicated that exposure of spermatozoa in *G*. *gemineoa* to both near-future (ΔpH– 0.3) and far-future (ΔpH– 0.5) ocean acidification scenarios would result in dramatically reduced sperm motility and swimming velocity.

To utilise sperm function effectively in *G*. *gemineoa* in monitoring water quality, a more comprehensive understanding of the male reproductive system is essential. Polychaetes exhibit a large variety of reproductive characteristics, which are reflected not only by their highly diverse methods of fertilisation, but also by the varied morphology of their spermatozoa [15, 16]. To date, a wide range of polychaetes have had their spermiogenesis, particularly the morphology of their spermatozoa, studied at the ultrastructural level. Spermatozoa in polychaetes have been categorised into three different types: ect-aquasperm, ent-aquasperm, and introsperm [17]. Ect-aquasperm, which is the most common sperm type among polychaetes, are liberated freely into the water column where they fertilise similarly released oocytes [15, 18]. This type of spermatozoa generally consists of a short acrosome, a spherical to oval nucleus, a short midpiece containing four to six mitochondria, and a flagellum with the typical 9 × 2 + 2 arrangement of microtubules [17]. Distinct from ect-aquasperm, both ent-aquasperm and introsperm are transferred to females *via* spermatophores, and oocytes are fertilised internally in the polychaete tube. While ent-aquasperm still spend a certain period of time in the water column, introsperm are completely isolated from the ambient aqueous environment when being transferred from male to female [16, 19]. On the basis of the external fertilisation strategy that *G*. *gemineoa* adopts and the primitive sperm morphology, the spermatozoa in this species have been identified as ect-aquasperm [17].

According to previous studies from our laboratory, the entire process of spermiogenesis in *G*. *gemineoa* occurs while the gametes are floating freely in the lumen of the germinal chamber unattached to nurse cells of any kind [20]. Such independence from supporting cells makes spermiogenesis in *G*. *gemineoa* a convenient model to uncover the mechanisms underlying this complex differentiation process. However, the normal pattern of spermatid differentiation remains unknown in this species. In the present article, the pattern of spermiogenesis in *G*. *gemineoa* has been described in detail at the ultrastructural level and a preliminary analysis of spermatid differentiation *in vitro* has been undertaken.

## Materials and methods

### Sample collection and maintenance

Aggregations of *G*. *gemineoa* were collected freshly at low tides between March and June from intertidal rock revetments at Merewether beach (32°56’34”S, 151°45’9”E), Merewether, New South Wales, Australia. The collection of this material was performed in accordance with NSW State legislature covering the collection of non-endangered and non-protected invertebrate species from the marine environment. The tubeworms were transported to the laboratory within 1 h of collection and were maintained in aerated polyethylene buckets with natural seawater obtained from the collection site. Samples were reared at constant room temperature of 20 ± 2°C and exposed to a 12-h light/12-h dark illumination cycle. All samples processed during this study were fixed within 24 h of collection.

### Transmission electron microscopy

Male adults were carefully extracted from their calcareous tubes with fine forceps and immediately immersed in 2.5% (v/v) glutaraldehyde (ProSciTech, Queensland, Australia) in artificial seawater (28.32 g NaCl, 0.77 g KCl, 5.41 g MgCl_2_∙6H_2_O, 7.13 g MgSO_4_∙7H_2_O, 1.18 g CaCl_2_ and 0.2 g NaHCO_3_ in 1 L distilled water) with an osmolality of 1,140 mOsm/kg and pH at 7.4 at room temperature for 1 h. The abdomen of each worm was cut into cubes no more than 1 mm^3^ in size. The specimens were then transferred to freshly made fixative and fixed at 4°C overnight. Fixed specimens were washed and post-fixed in 1% (v/v) osmium tetroxide in artificial seawater (pH 7.4) for 2 h. After dehydration through an ascending acetone series, the specimens were infiltrated with 50% (v/v) Spurr’s resin embedding medium (ProSciTech, Queensland, Australia) in absolute acetone on a rotator at room temperature overnight. The resin medium was then replaced with 100% Spurr’s resin and the specimens were infiltrated for a further 12 h. The specimens were then embedded in freshly made 100% Spurr’s resin in polyethylene embedding capsules or moulds (ProSciTech, Queensland, Australia) and polymerised in an oven at 65°C for 24 h. Resin blocks were cut into semi-thin sections on a Reichert Ultracut S Microtome (Leica, Wien, Austria) with freshly made glass knives or a diamond knife (Diatome, Bienne, Switzerland). Semi-thin sections were stained with 0.5% (w/v) toluidine blue (BDH Chemicals, Kilsyth, Australia) in absolute ethanol and observed under the Zeiss Axiovert S100 microscope (Carl Zeiss, Oberkochen, Germany). Ultra-thin sections at a thickness of 60 nm were cut and stained with aqueous saturated uranyl acetate and Reynolds’ lead citrate (BDH Chemicals, Poole, England) and examined under a JEM-2100EX II transmission electron microscope (JEOL, Tokyo, Japan) at 80 kV.

### *In vitro* culture of spermatids

Adult male *G*. *gemineoa* were extracted from their calcareous tubes and immediately immersed in filtered natural seawater. The worms spontaneously released their spermatozoa on being exposed to seawater. They were allowed to sit for at least 10 min until the spawning ceased. After that, the worms were rinsed vigorously in seawater and transferred to clean seawater. More spermatozoa, along with a considerable number of spermatids, were stimulated to release by puncturing the abdominal wall with a syringe needle and gently pressing the abdomen with a pair of forceps. To enrich the spermatids, 1.5 mL of the sperm suspension collected from at least 10 male adults was applied to the top of a discontinuous Percoll gradient (10%, 20%, 30% and 40% Percoll in artificial seawater, each layer comprising 1.5 mL) in a 15 mL Falcon tube and centrifuged at 500 × g for 20 min. The centrifugation process separated the cells into two distinct bands: an upper thin band containing spermatids at various differentiation stages and a lower thick bank containing spermatozoa. The thin band was carefully transferred to a 1.5 mL Eppendorf tube by using transfer pipettes. The Percoll was then eliminated by centrifugation at 500 × g for 5 min and the resultant pellet containing mostly spermatids was resuspended in culture medium.

The isolated spermatids were cultured in male germinal fluid, boiled male germinal fluid, female germinal fluid, and 10% foetal bovine serum (FBS; Sigma Aldrich, Castle Hill, Australia) in RPMI 1640 media (Thermo Fisher Scientific, North Ryde, Australia) to test their capacity to induce the differentiation of spermatids *in vitro*. Osmolality was adjusted to that of the male germinal fluid (1,135 mOsmol/kg) with artificial seawater.

To obtain the male and female germinal fluids, adult worms were extracted from their tubes and placed on a dry Petri dish. To stimulate the release of germinal fluid, the abdominal wall was lightly punctured with a 30G × 0.5-inch syringe needle and then gently pressed with a pair of forceps. During this process the digestive tract and the main artery were not disturbed in order to avoid contamination by their contents. The germinal fluid was collected by using a Hamilton^®^ 7000.5KHs 0.5 μL syringe (Sigma Aldrich, Castle Hill, Australia) and pooled together into a 1.5 mL Eppendorf tube. About 20 to 30 adult worms were used for each attempt at cell culture so that at least 100 μL of germinal fluid could be obtained. The germinal fluid was then centrifuged at 19,083 × g for 5 min and the supernatant was transferred to a clean Eppendorf tube. 1 μL of purified spermatids and 1 μL of 10,000 U/mL Penicillin-Streptomycin (Thermo Fisher Scientific, North Ryde, Australia) were added to 98 μL of the male or female germinal fluid and mixed thoroughly. By using a Pasteur pipette, the purified spermatids in the germinal fluid were immediately transferred to a microscope side chamber made with a 40 mm × 22 mm HybriWell™ hybridization sealing chamber (Thermo Fisher Scientific, North Ryde, Australia). To minimise evaporation of the culture medium, the two ports of the slide chamber were sealed firmly by two seal-tabs.

The sperm cells were cultured inside a Zeiss microscopic physiological chamber (Carl Zeiss, Oberkochen, Germany) with 5% CO_2_ and 100% humidity air at 21°C for up to 36 h. The slide chamber was kept in the dark by wrapping the physiological chamber with aluminium foil. The cultured spermatids were examined under a Zeiss Axiovert S100 microscope (Carl Zeiss, Oberkochen, Germany) equipped with a Zeiss AxioCam MRm camera (Carl Zeiss, Oberkochen, Germany) at 0 h, 12 h, 24 h and 36 h of incubation. The percentages of spermatids at different stages were counted from a hundred randomly selected cells.

### Statistical analysis

All data presented in this article were generated from at least three biological replicates to meet the requirements for statistical validity. The measurements of spermatids and their subcellular organelles are all presented as mean ± SD, which were calculated from at least 20 samples from each step of spermatid development. The statistical difference between spermatids at different developmental stages was determined using the Student’s t-test or ANOVA depending on whether paired or multiple comparisons were being made. Differences were recognised as statistically significant if the *P* value was less than 0.05. All statistical analyses were performed using Microsoft Excel 2013 (Microsoft Corporation, Redmond, USA).

## Results

Spermatids have been classified into ten steps based on the morphological development of the acrosome, nucleus, flagellum and other organelles at the ultrastructural level.

### Step 1 spermatids

During the second meiotic division of spermatogenesis, each secondary spermatocyte divided into a pair of step 1 spermatids, which were interconnected by a common cytoplasmic bridge. At this stage of development, these spermatids became detached from the seminiferous epithelium to float freely in the lumen of the germinal chamber.

**Fig 1:**
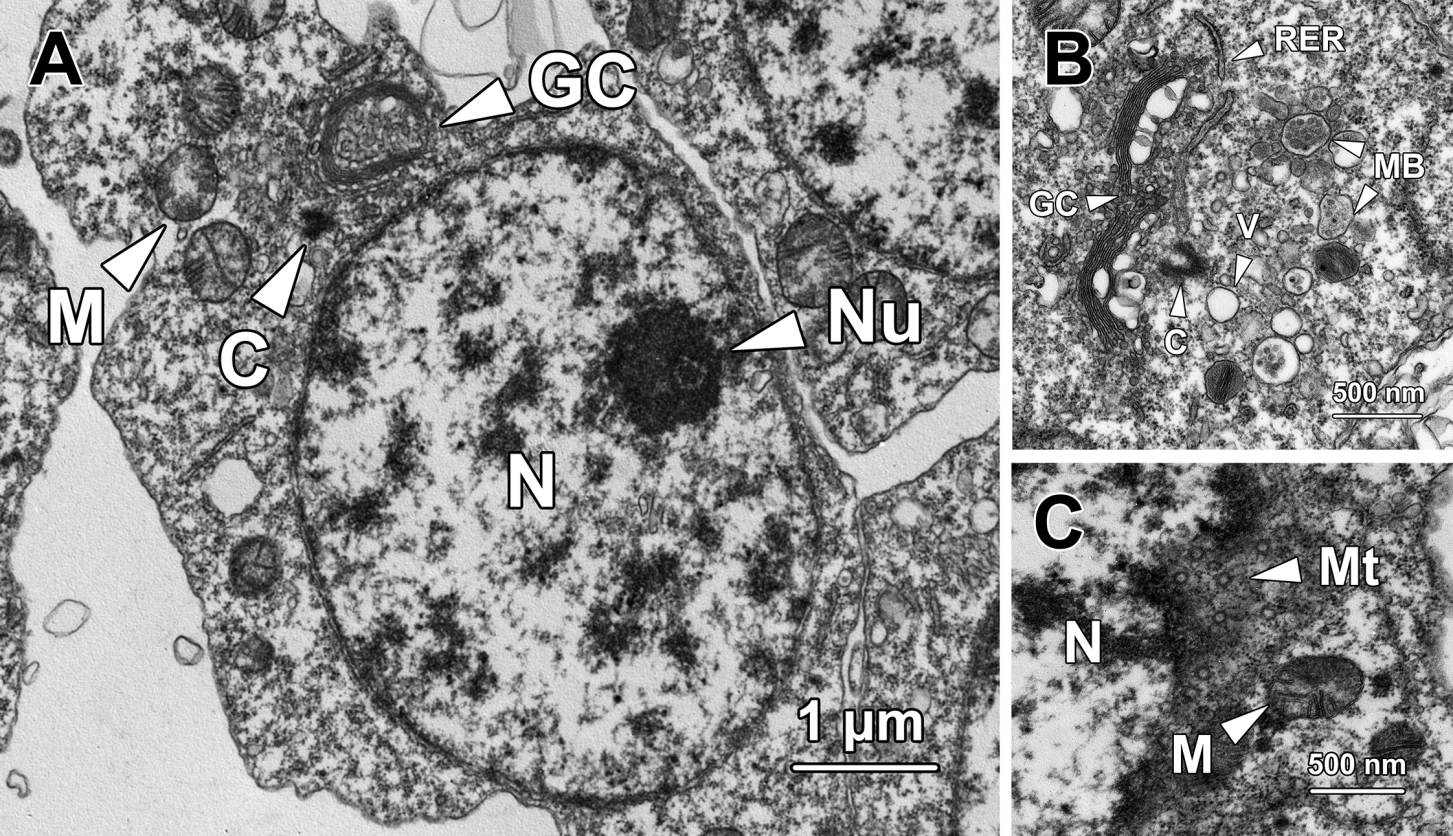
Transmission electron micrographs of step 1 spermatids. **(A)** Step 1 spermatids are ovoid with an oval nucleus (N) containing one or two eccentric nucleoli (Nu) and a large number of randomly dispersed small patches of chromatin. A well-developed Golgi complex (GC) is found in close proximity to the nucleus. A pair of centrioles (C) resides adjacent to both the Golgi complex and the nuclear membrane. Spherical mitochondria (M) are in clusters in the cytoplasm. **(B)** Numerous small membrane-bound vesicles (V) and multivesicular bodies (MB) are presented at the concave surface of the Golgi complex (GC). Centrioles (C) are also found next to the Golgi complex. Rough endoplasmic reticulum (RER) is randomly distributed in the cytoplasm. **(C)** Bundles of microtubules (Mt) are found tightly surrounding the nucleus (N), forming a circular manchette. M, mitochondria.

Step 1 spermatids were ovoid in shape with an average cellular diameter of 6.4 ± 0.46 μm (Fig 1A). The nucleus (N) was spherical- to oval-shaped and approximately 4.38 ± 0.46 μm in diameter. Small patches of dense heterochromatin were randomly scattered in the nucleoplasm and lined the inner nuclear membrane. Euchromatin was sparse and evenly distributed throughout the nucleoplasm. The nucleus contained one or two prominent eccentric nucleoli (Nu).

In the perinuclear region of the cytoplasm, a well-developed Golgi complex (GC) was observed, with a large number of secretory small membrane-bound vesicles (V) and multivesicular bodies (MB) at its concave surface (Fig 1B). These vesicles later fused with each other to form a proacrosomal vacuole. A pair of centrioles (C) was located in close proximity to both the nuclear membrane and the Golgi complex (Fig 1A). Spherical-shaped mitochondria (M) with an average diameter of 0.44 ± 0.06 μm were clustered near the Golgi complex (Fig 1A). Rough endoplasmic reticulum (RER) was randomly distributed throughout the whole cytoplasm (Fig 1B). Bundles of microtubules (Mt) surrounded the nucleus and formed the circular manchette that facilitated shaping of the nucleus (Fig 1C).

### Step 2 spermatids

**Fig 2:**
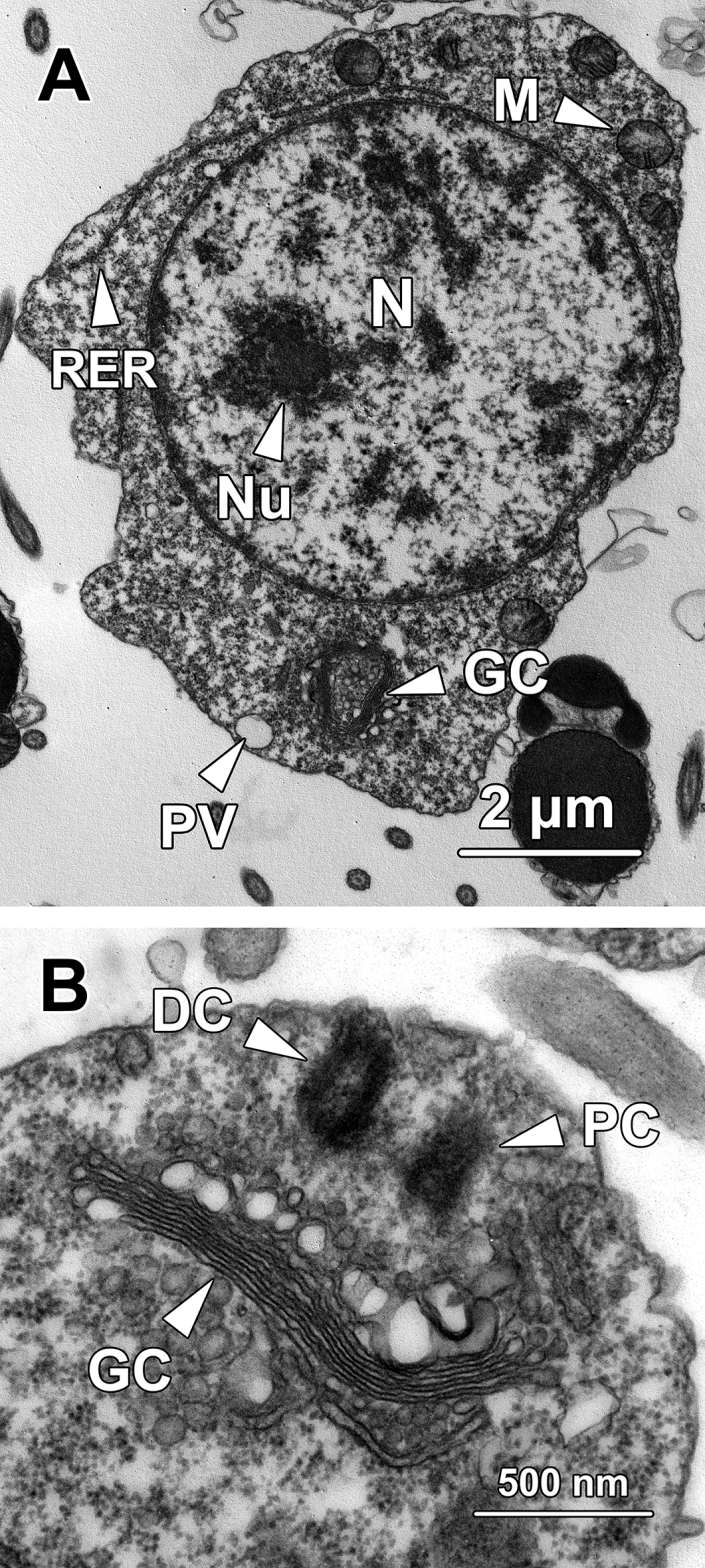
Transmission electron micrographs of step 2 spermatids. **(A)** Step 2 spermatids are oval with a spherical to oval nucleus (N) containing an eccentric nucleolus (Nu) and small clumps of chromatin. In the periphery of the nucleus, a distinct Golgi complex (GC) is observed adjacent to a spherical proacrosomal vacuole (PV) with an electron-lucent matrix, which originates from the fusion of small membrane-bound vesicles. Continuous profiles of rough endoplasmic reticulum (RER) are found running around the nucleus. Spherical mitochondria (M) are randomly dispersed throughout the cytoplasm. **(B)** The two centrioles in step 2 spermatids remain in close proximity to the Golgi complex (GC) but become relatively distant from the nucleus. While the distal centriole (DC) approaches the plasma membrane, the proximal centriole (PC) orientates parallel to the former and is relatively distant from the cell membrane.

Step 2 spermatids remained oval-shaped with a spherical to oval nucleus (Fig 2A). The average cellular and nuclear diameters of step 2 spermatids were 5.89 ± 0.41 μm and 4.15 ± 0.42 μm respectively. While their cellular size was significantly lower than that of step 1 spermatids (P < 0.001), the nuclear size had no significant difference from that of their immediate precursors (P > 0.05). Dense clumps of chromatin were randomly dispersed throughout the nucleus and also lined the inner nuclear membrane. A conspicuous nucleolus (Nu) was eccentrically situated in the nucleoplasm.

Step 2 spermatids were characterised by the formation of the proacrosomal vacuole (PV), which was generated by coalescence of the small membrane-bound vesicles secreted by the Golgi complex (GC). The proacrosomal vacuole at this stage was spherical with a nearly electron-lucent matrix (Fig 2A). The two centrioles migrated away from the nuclear membrane and gradually approached the cytoplasmic membrane, during which the two centrioles were orientated parallel instead of perpendicular to each other. The centriole in close association with the plasma membrane was the distal centriole (DC), which later gave rise to the sperm flagellum; the centriole relatively distant from the plasma membrane was the proximal centriole (PC; Fig 2B). Spherical-shaped mitochondria (M) were randomly dispersed around the periphery of the nucleus while continuous profiles of rough endoplasmic reticulum (RER) tightly surrounded the nucleus (Fig 2A).

### Step 3 spermatids

**Fig 3:**
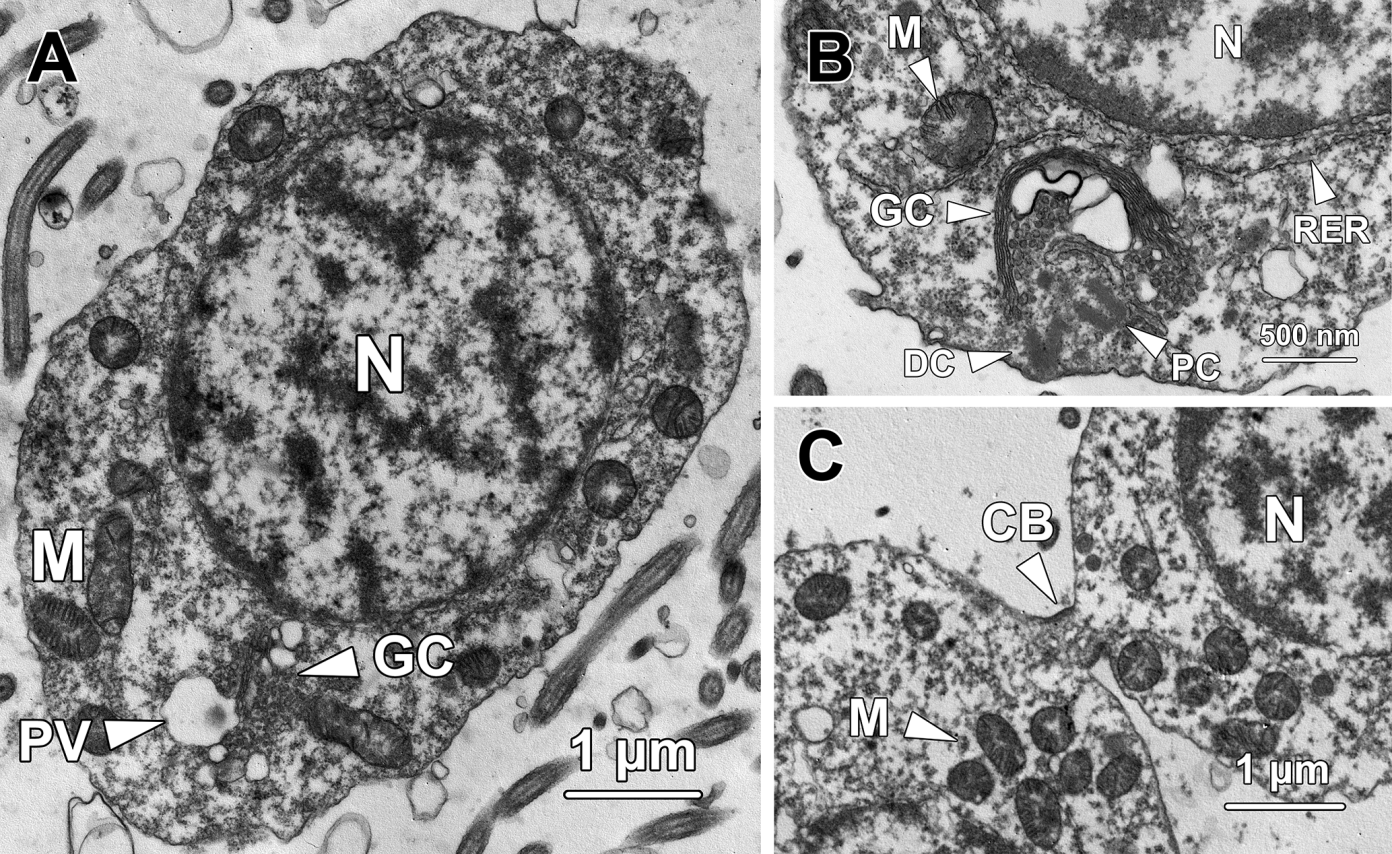
Transmission electron micrographs of step 3 spermatids. **(A)** Similar to their immediate precursors, step 3 spermatids remain ovoid with a spherical to oval nucleus (N) containing rod-shaped chromatin blocks. Thick patches of chromatin also attach to the inner nuclear membrane. Next to the Golgi complex (GC), a spherical proacrosomal vacuole (PV) is observed containing a patch of electron-dense material. A portion of mitochondria (M) transform into rod-shape organelles in step 3 spermatids. **(B)** Both the proximal (PC) and distal centrioles (DC) remain in close proximity to the Golgi complex (GC) and start to orientate perpendicularly to each other. Rough endoplasmic reticulum (RER) is found surrounding the nucleus (N). M, mitochondria. **(C)** The step 3 spermatids are in pairs and interconnected by a cytoplasmic bridge (CB). Mitochondria (M) are clustered at the cytoplasm near the cytoplasmic bridge. N, nucleus.

Step 3 spermatids had a cellular diameter of 6.01 ± 0.51 μm and a nuclear diameter of 3.87 ± 0.45 μm, which were similar to that of the step 2 spermatids (P > 0.05). Distinct from the irregular-shaped small chromatin blocks in the nucleoplasm of step 1 or 2 spermatids, the chromatin blocks in step 3 spermatids exhibited a rod-like appearance. The chromatin that clumped against the inner nuclear membrane became thicker than that in step 2 spermatids (Fig 3A).

The proacrosomal vacuole (PV) remained adjacent to the Golgi complex (GC) but became larger as more Golgi-derived vesicles fused with it. At this stage, a small patch of electron-dense material formed eccentrically in the poorly stained matrix of the proacrosomal vacuole (Fig 3A). Although both of the two centrioles remained close to the Golgi complex, the distal centriole (DC) docked onto the plasma membrane, where the flagellum would subsequently form. The proximal centriole (PC) oriented almost perpendicularly to the distal centriole (Fig 3B). Different from the mitochondria in younger spermatids, which were all spherical in shape, a portion of the mitochondria (M) in step 3 spermatids exhibited a rod-shaped appearance (Fig 3A); the long axis was about 0.91 ± 0.16 μm and short axis was averagely 0.38 ± 0.13 μm. Noticeably, the long axis of these mitochondria was approximately twice as long as the diameter of the spherical-shaped mitochondria, whereas the short axis had no significant difference from the diameter of spherical mitochondria (P > 0.05). At this step, the spermatids remained in pairs and were interconnected by a cytoplasmic bridge (CB; Fig 3C).

### Step 4 spermatids

**Fig 4:**
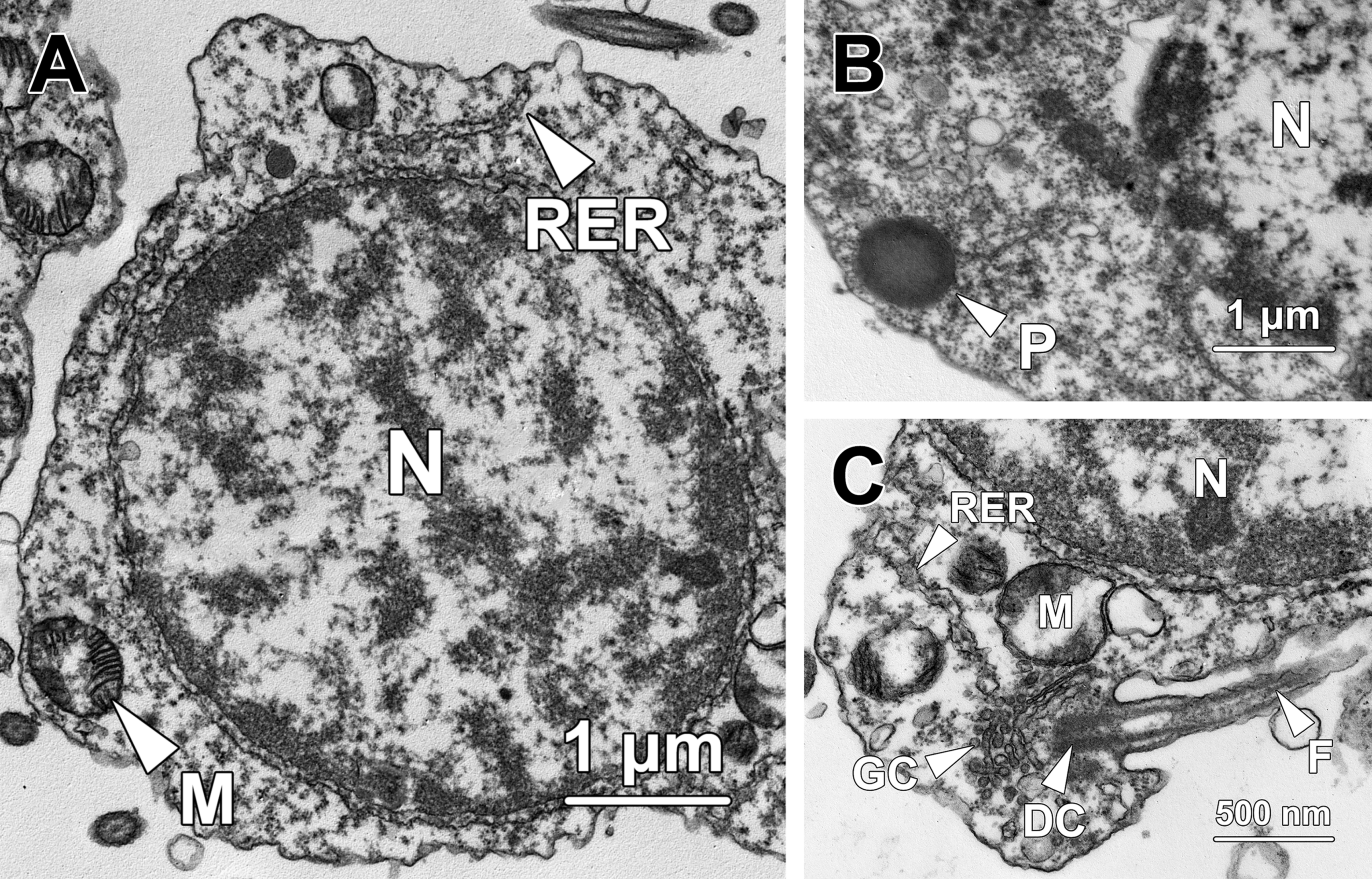
Transmission electron micrographs of step 4 spermatids. **(A)** Chromatin blocks in the nucleus (N) of step 4 spermatids remain rod-shaped but become sparser than those in previous steps, indicating that chromosome decondensation commences at this stage. Mitochondria (M) and rough endoplasmic reticulum (RER) are randomly distributed throughout the cytoplasm. **(B)** The proacrosomal vacuole transforms into a proacrosome (P) in step 4 spermatids, with its matrix being mostly occupied by electron-dense material. N, nucleus. **(C)** The distal centriole (DC) next to the Golgi complex (GC) forms a flagellum (F) protruding out of step 4 spermatids. N, nucleus; RER, rough endoplasmic reticulum.

Step 4 spermatids were spherical in shape containing a spherical nucleus (Fig 4A). They exhibited similar cellular and nuclear sizes to those of step 3 spermatids (P > 0.05). The average cellular and nuclear diameters of step 4 spermatids were 5.7 ± 0.45 μm and 4.04 ± 0.5 μm respectively. The chromatin blocks remained rod-shaped, but were sparser compared with those in step 3 spermatids, indicating the commencement of chromosome decondensation in step 4 spermatids.

Step 4 spermatids were characterised by the formation of proacrosome and flagellum. The patch of electron-dense material in the proacrosomal matrix (Fig 3B) expanded and eventually occupied the majority of the proacrosomal matrix, thereby transforming the proacrosomal vacuole into a proacrosome (P; Fig 4B). Meanwhile, the proacrosome came into closer proximity with the plasma membrane than step 3 spermatids. The distal centriole (DC) which resided near the Golgi complex (GC) protruded out of the cytoplasm to form a flagellum (F; Fig 4C).

### Step 5 spermatids

**Fig 5:**
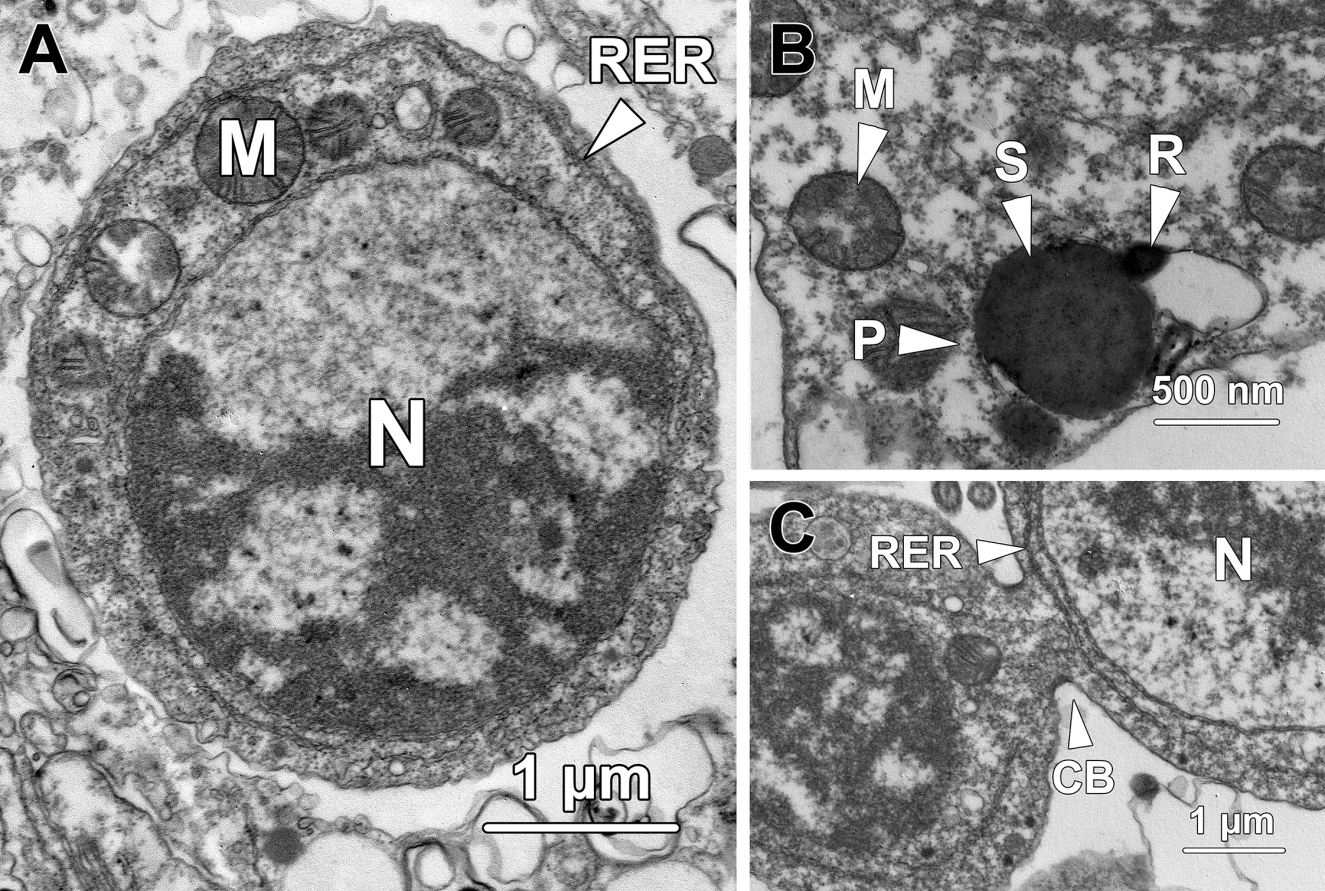
Transmission electron micrographs of step 5 spermatids. **(A)** As the nuclear size (N) of step 5 spermatids decreases, the decondensed chromosomes are aggregated into a retiform pattern. Large spherical mitochondria (M) are observed gathering at one end of the spermatids. RER, rough endoplasmic reticulum. **(B)** In the proacrosome (P) of step 5 spermatids, a ring-shaped structure (R) forms surrounding the sphere (S) at the centre. M, mitochondria; N, nucleus. **(C)** Step 5 spermatids remain interconnected by a cytoplasmic bridge (CB). A continuous profile of rough endoplasmic reticulum (RER) is found surrounding the nucleus.

Step 5 spermatids had a cellular diameter of 4.81 ± 0.64 μm and a nuclear diameter of 3.35 ± 0.51 μm, both of which were significantly smaller than those of step 4 spermatids (P < 0.01). The chromosomes were further decondensed and transformed into a retiform pattern at this stage (Fig 5A).

The proacrosome in step 5 spermatids transformed from a simple sphere into its mature form, a ring-shaped deposit of electron-dense material (R) surrounding the sphere (S) at the centre, which appeared as two small electron-dense spheres residing at each side of a large sphere in longitudinal section (Fig 5B). A small number of extremely large mitochondria (M) with a diameter of over 500 nm clustered at one end of the spermatid (Fig 5A). These larger spherical-shaped mitochondria later formed the mitochondrial sheath surrounding the midpiece of the sperm flagellum. Step 5 spermatids were still in pairs and interconnected by a cytoplasmic bridge (CB; Fig 5C).

### Step 6 spermatids

**Fig 6:**
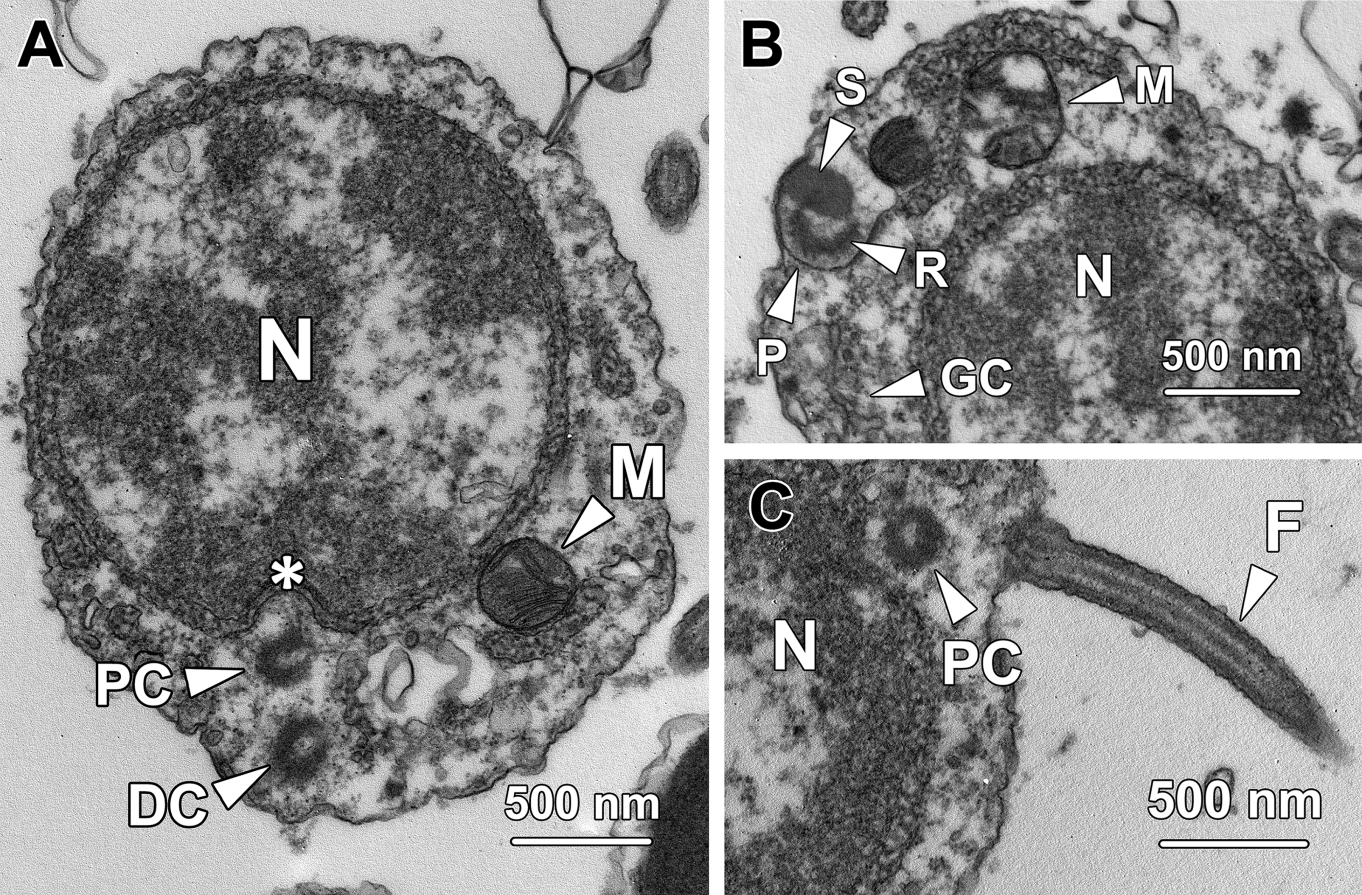
Transmission electron micrographs of step 6 spermatids. **(A)** An implantation fossa (*), which appears as a shallow depression, forms at the posterior end of the nucleus (N). The proximal centriole (PC) is in close proximity to the implantation fossa while the distal centriole remains docked with the cytoplasmic membrane. Large spherical-shaped mitochondria (M) migrate towards the pole where the centrioles reside. **(B)** This electron micrograph represents a cross-section through a small portion of the proacrosome, showing fragments of both the ring-shaped structure (R) and the central sphere (S). The proacrosomal matrix becomes being occupied by coarse granules. A portion of the proacrosome adheres to the cytoplasmic membrane. Large spherical mitochondria are found next to the proacrosome. N, nucleus. **(C)** As the proximal centriole (PC) embeds into the implantation fossa, the flagellum (F) extends from the distal centriole and is further elongated. N, nucleus.

Compared with step 5 spermatids, both the cellular and nuclear diameters decreased significantly in step 6 spermatids, generating values of 2.81 ± 0.25 μm (P < 0.01) and 2 ± 0.18 μm (P < 0.001) respectively. The chromatin continued to dencondense and transformed into larger chromatin blocks that occupied almost half the volume of the entire nucleoplasm (Fig 6A).

In step 6 spermatids, the proacrosome still appeared as a spherical vacuole containing a central sphere (S) and a ring-like structure (R). The proacrosomal matrix, which was electron-lucent in step 3 spermatids, started to be occupied by coarse granules. The proacrosome further approached the plasma membrane, with its membrane firmly adhering to this structure (Fig 6B). In step 6 spermatids, a shallow depression formed at the posterior pole of the nucleus, which was the implantation fossa (*) for the proximal centriole (Fig 6A). While the proximal centriole gradually came to lie in close proximity to the implantation fossa, the formative flagellum (F) originated from the distal centriole (DC) and was further elongated (Fig 6C). The formation of the implantation fossa marked the posterior end of the cell, where the proximal centriole and flagellum resided and the mitochondrial sheath later formed. At this stage, the large spherical-shaped mitochondria (M) were still situated away from the centrioles (Fig 6A).

### Step 7 spermatids

**Fig 7:**
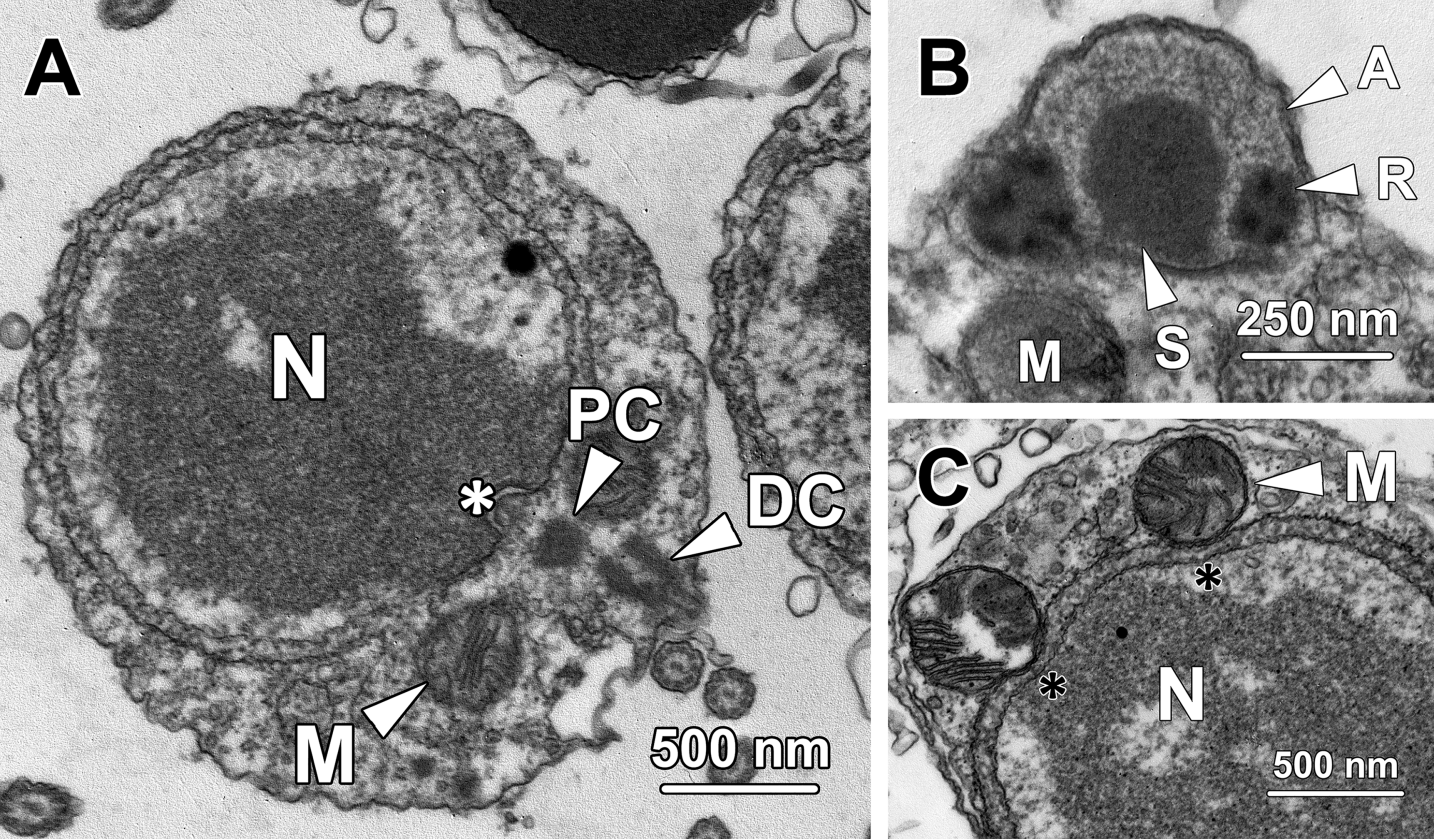
Transmission electron micrographs of step 7 spermatids. **(A)** The chromatin in the nucleus of step 7 spermatids exhibits a coarse granular form and aggregates into a large irregular-shaped block. The chromatin block attached to the posterior end of the nucleus (N), where an implantation fossa (*, white asterisk) has formed. The proximal centriole (PC) remains in close proximity to the fossa and orientates perpendicularly to the distal centriole (DC). The large spherical-shaped mitochondria (M) become surrounded the centrioles and start to form the mitochondrial sheath at the base of the flagellum. **(B)** The acrosome (A) transforms into an inverted-bowl shape with its anterior region tightly adherent to the cell membrane. In the acrosomal matrix, both the central sphere (S) and the ring-shaped structure (R) attach to the inner posterior membrane of the acrosome. The acrosome is located adjacent to large spherical mitochondria (M) which will ultimately form the mitochondrial sheath of the flagellum. **(C)** Where the mitochondria (M) approach the nuclear membrane (N), the nuclear envelope curves inward to form depressions (*, black asterisks).

The nuclei of step 7 spermatids were spherical and contained an irregular-shaped chromatin block with a coarse granular appearance (Fig 7A). The large heterochromatin block, which attached to the posterior pole of the inner nuclear membrane, occupied the majority of the nucleoplasm and the remaining portion of the nucleus was occupied by sparse euchromatin.

In step 7 spermatids, the acrosome (A) transformed from the spherical into an inverted-bowl or hemispherical shape (Fig 7B). The anterior membrane of the acrosome was arc-shaped and fully attached to the plasma membrane. The posterior membrane of the acrosome was relatively flat and in close proximity to the large spherical mitochondria (Fig 7B), indicating the acrosome was located next to the flagellum at this step. In the acrosome, both the central sphere (S) and the ring-shaped structure (R) were situated close to the posterior membrane but relatively distant from the anterior membrane. Noticeably, a small number of electron-dense particles lined the interior border of the ring. The acrosomal matrix at this step was completely filled with coarse granules (Fig 7B).

In step 7 spermatids, the large spherical-shaped mitochondria translocated to the pole where the centrioles resided and started to form the mitochondrial sheath surrounding the base of the flagellum (Fig 7A). Simultaneously, the mitochondria gradually approached the nucleus and the corresponding portions of nuclear membrane curved inward to form a concave recess (*, black asterisks) to accommodate these mitochondria (Fig 7C).

### Step 8 spermatids

**Fig 8:**
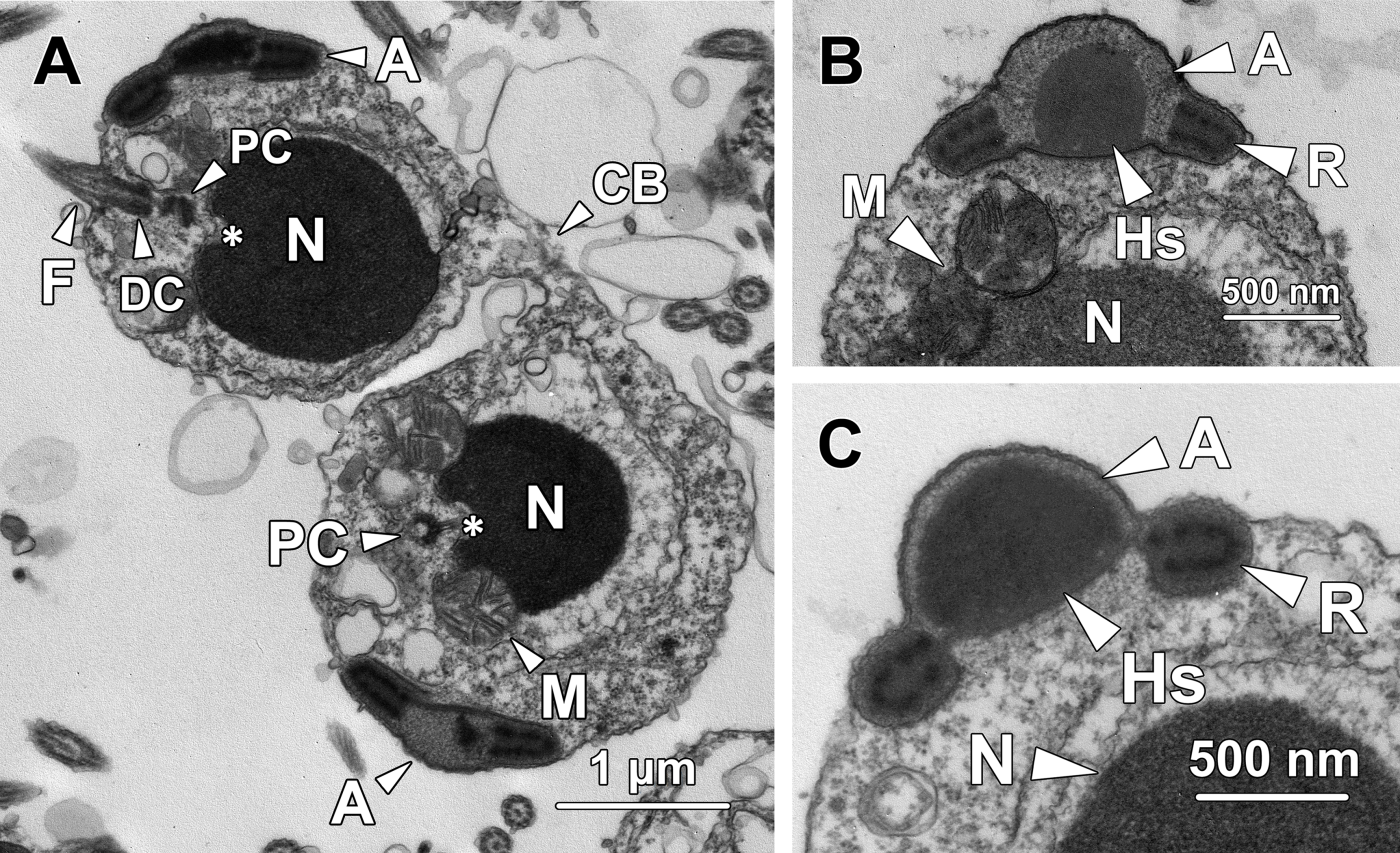
Transmission electron micrographs of step 8 spermatids. **(A)** Step 8 spermatids are still interconnected by a cytoplasmic bridge (CB). The nucleus (N) remains spherical in shape while the chromatin block transforms into an oval shape with a highly condensed homogeneous appearance. The proximal centriole (PC) extends its microtubules into the implantation fossa (*) and orientates perpendicularly to the flagellum (F) which originates from the distal centriole (DC). The developing acrosome (A) remains adjacent to the flagellum at this stage. **(B)** The central sphere firmly attaches to the posterior membrane of the acrosome (A) and transforms into a hemisphere (Hs). The ring-like structure (R), with darker particles lining its interior border, also comes into direct contact with the acrosomal membrane on both sides. The acrosomal matrix is filled with densely packed coarse granules. M, mitochondria; N, nucleus. **(C)** As the morphogenesis of the acrosome proceeds, the central hemisphere (Hs) increases its volume and ascends towards the anterior membrane, resulting in the formation of an indentation at the posterior end of the acrosome. N, nucleus; R, ring-shaped structure.

Spherical-shaped step 8 spermatids were still in pairs and interconnected by a cytoplasmic bridge (CB; Fig 8A). The nucleus remained roughly spherical but the chromatin block transformed into an oval shape. The chromatin gradually condensed from a coarse granular form (Fig 8B) to a dense homogeneous granular form (Fig 8A and 8C). The remaining portion of the nucleoplasm was occupied by sparse euchromatin.

The acrosome of step 8 spermatids remained adjacent to the mitochondria and flagellum at the posterior pole of the cytoplasm (Fig 8A). In the acrosome, the central sphere transformed into a hemisphere (Hs) the flat side of which was firmly attached to the inner posterior membrane (Fig 8B). The ring-like structure (R), which was interiorly lined with conspicuous darker particles, also came in direct contact with the acrosomal membrane on both sides (Fig 8B). The coarse granules that occupied the entire acrosomal matrix became denser than those of step 8 spermatids. As differentiation proceeded, the central hemisphere (Hs) increased its volume and gradually occupied the whole acrosomal matrix (Fig 8B to 8C). Simultaneously, the central hemisphere, along with the adherent portion of posterior membrane, moved anteriorly, resulting in the formation of a shallow cylinder-shaped indentation at the posterior pole of the acrosome (Fig 8C). Noticeably, the posterior membrane adjacent to the hemisphere was lined with a thin band of dense granular material, which later would become the subacrosomal material (Fig 8B and 8C).

The proximal centriole (PC) in step 8 spermatids remained perpendicular to the flagellum (F) and continued the implantation process by extending its microtubules into the implantation fossa (*, see the lower spermatid in Fig 8A). The spherical mitochondria firmly adhered to the concave surface at the posterior end of the nucleus and formed the mitochondrial sheath that surrounded the proximal centriole and the flagellum (Fig 8A).

### Step 9 spermatids

**Fig 9:**
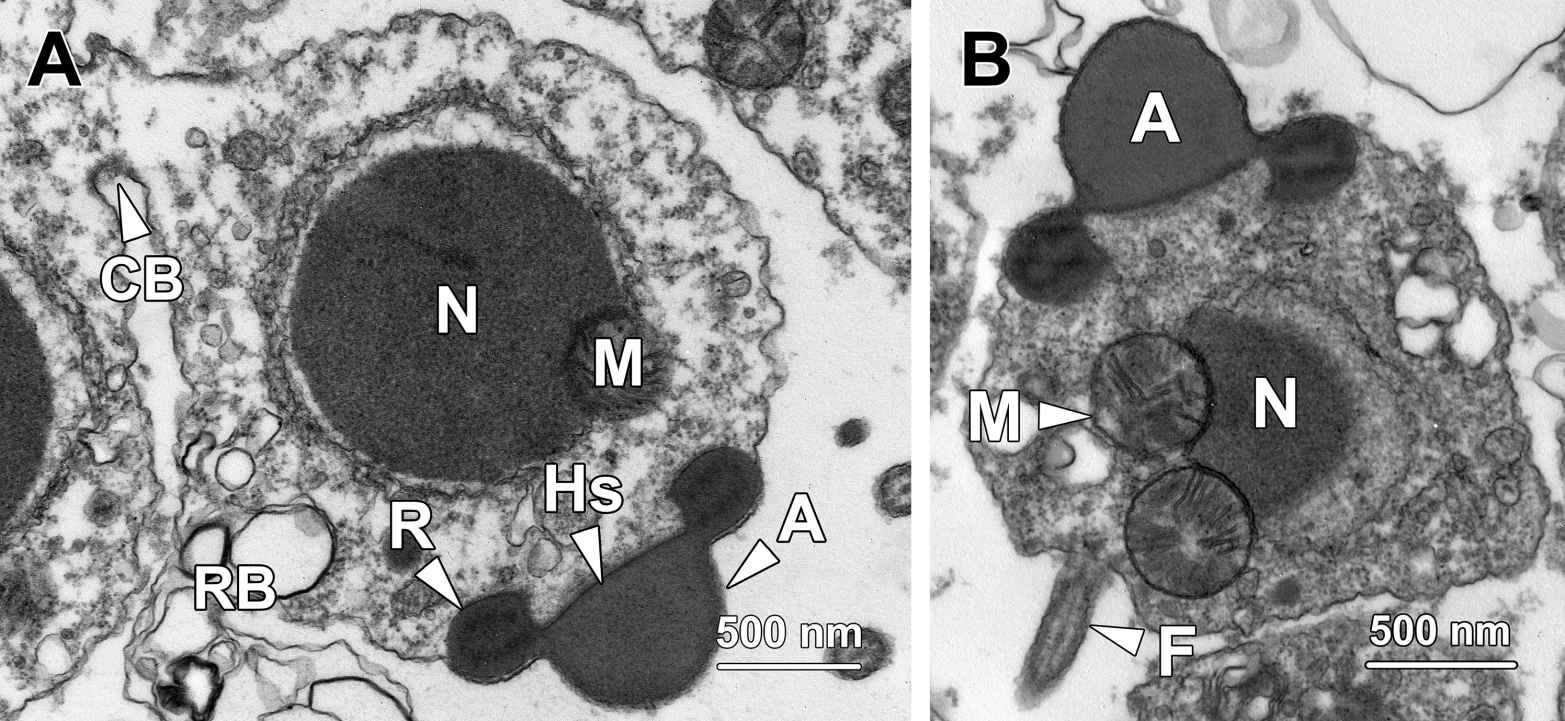
Transmission electron micrographs of step 9 spermatids. **(A)** Step 9 spermatids are still in pairs and interconnected by a cytoplasmic bridge (CB). The central hemisphere (Hs) in the acrosome occupies the entire acrosomal matrix and connects to the ring-like structure (R) on both sides. The whole acrosome exhibits an identical electron density except for the darker particles lining the interior border of the ring structure. Multiple irregular-shaped residual bodies (RB) containing excess cytoplasm appear in step 9 spermatids. M, mitochondria; N, nucleus. **(B)** In step 9 spermatids, the acrosome (A) starts to migrate towards the anterior end of the cytoplasm, which is opposite to the pole where the flagellum (F) and mitochondria (M) reside. The mitochondria remain firmly adherent to depressions at the posterior end of the nucleus (N).

Step 9 spermatids remained in pairs and were interconnected by a cytoplasmic bridge (Fig 9A). The chromatin in the nucleus accomplished its condensation and exhibited a homogeneous granular appearance.

Instead of residing adjacent to the flagellum at the posterior cytoplasm, the acrosome was found half way to the anterior region of step 9 spermatids (Fig 9B), suggesting that anterior migration of the acrosome commenced on completion of chromatin condensation. In the acrosome, the central hemisphere (Hs) further expanded and occupied the entire acrosomal matrix coming into direct contact with the ring-like structure at both sides (Fig 9A and 9B). The electron density was identical throughout the entire acrosome, except for the darker particles lining the interior border of the ring structure. Similar to that in step 8 spermatids, the posterior membrane of the acrosome was still externally lined with a thin band of dense granular material.

In step 9 spermatids, clusters of irregular-shaped vesicles were randomly dispersed in the cytoplasm, which formed the residual bodies (RB) containing excess cytoplasm that would be subsequently expelled from the spermatids (Fig 9A and 9B).

### Step 10 spermatids

**Fig 10:**
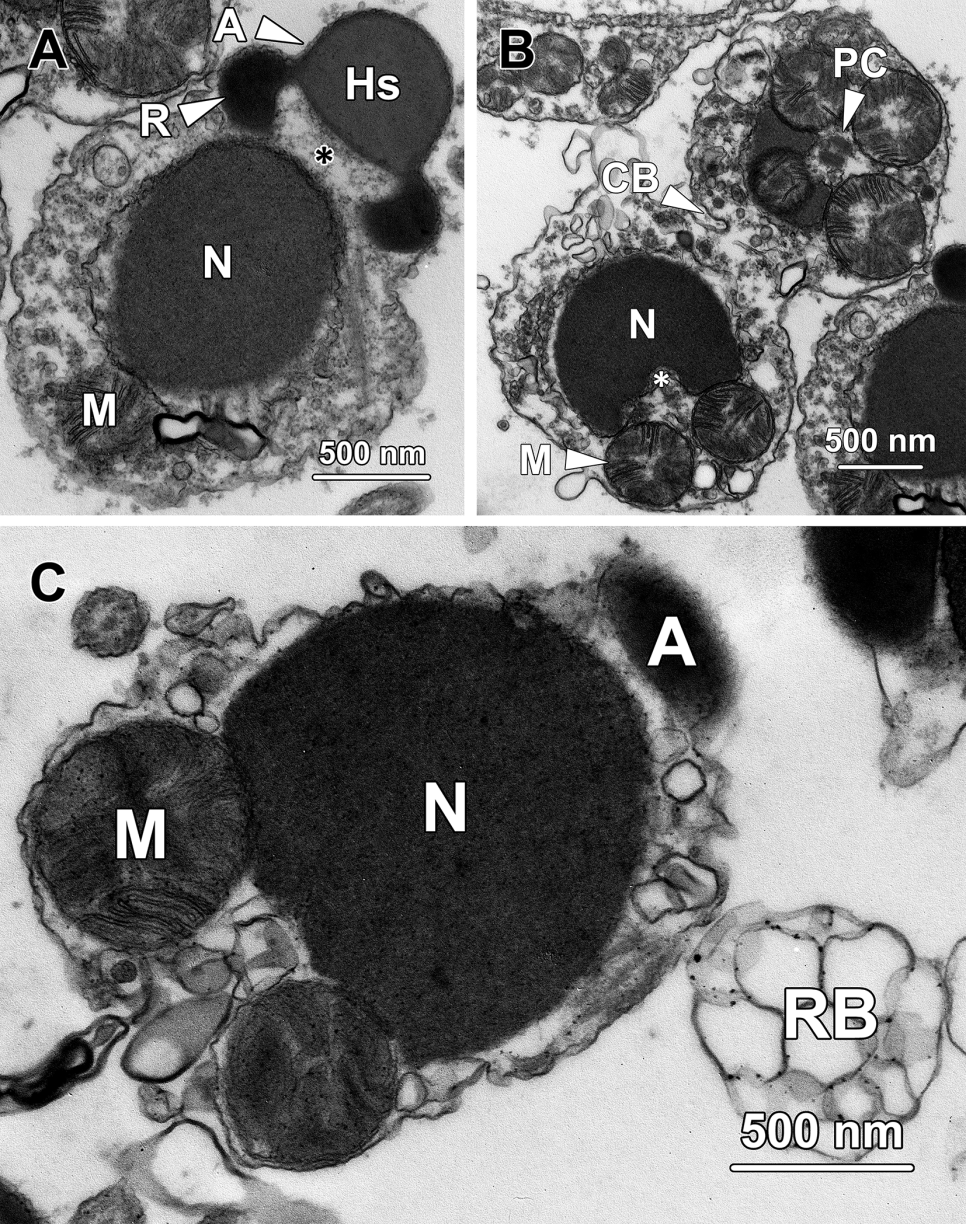
Transmission electron micrographs of step 10 spermatids. **(A)** The oval nucleus (N) is occupied by fully condensed homogeneous granular chromatin. The acrosome (A) reaches the anterior apex of the cytoplasm in step 10 spermatids, opposite to the pole where the mitochondria (M) and flagellum reside. As the dark particles spread throughout the ring-like structure, the ring appears darker than the central hemisphere. **(B)** Early step 10 spermatids remain in pairs and are interconnected by a cytoplasmic bridge (CB). The cytoplasm contains clusters of irregular-shaped vesicles, indicating the continuous formation and expulsion of residual bodies. The formation of mitochondrial sheath is accomplished, with four spherical mitochondria (M) surrounding the proximal centriole (PC). *, implantation fossa. **(C)** In late step 10 spermatids, numerous clusters of irregular-shaped vesicles are found inside and adjacent to the cytoplasm, suggesting the discharge of residual bodies (RB) occurs dramatically at this stage. With the elimination of excess cytoplasm, the cytoplasmic bridge connecting the spermatids disintegrates, resulting in the formation of two independent spermatozoa. A, acrosome; M, mitochondria; N, nucleus.

The nucleus of step 10 spermatids was oval and contained fully condensed chromatin with a homogeneous granular appearance (Fig 10A).

The acrosome reached the anterior pole of the cytoplasm, which was opposite to the midpiece and flagellum (Fig 10A). The entire acrosome was filled with homogeneous electron-dense material. The darker particles previously lining the interior border spread throughout the entire ring, resulting in the ring-shaped structure being darker than the hemisphere. Noticeably, the posterior end of the acrosome became closer to but never attached to the nuclear membrane, forming a subacrosomal space between the acrosome and the nucleus (Fig 10A). At this stage, the subacrosomal space only contained sparse granules and only the posterior membrane of the acrosome was lined with dense granular material. The formation of the mitochondrial sheath was also completed in step 10 spermatids, with four spherical mitochondria tightly surrounding the proximal centriole (PC; Fig 10B).

Noticeably, early step 10 spermatids were maintained in pairs connected by a cytoplasmic bridge (CB; Fig 10B). With the continuous expulsion of residual bodies containing excess cytoplasm, the cytoplasmic bridge collapsed and each pair of spermatids became two independent spermatozoa at the end of step 10 (Fig 10C). As a result, the cytoplasm in the spermatozoa was extremely limited and barely contained any ribosomes.

### Spermatozoa

**Fig 11:**
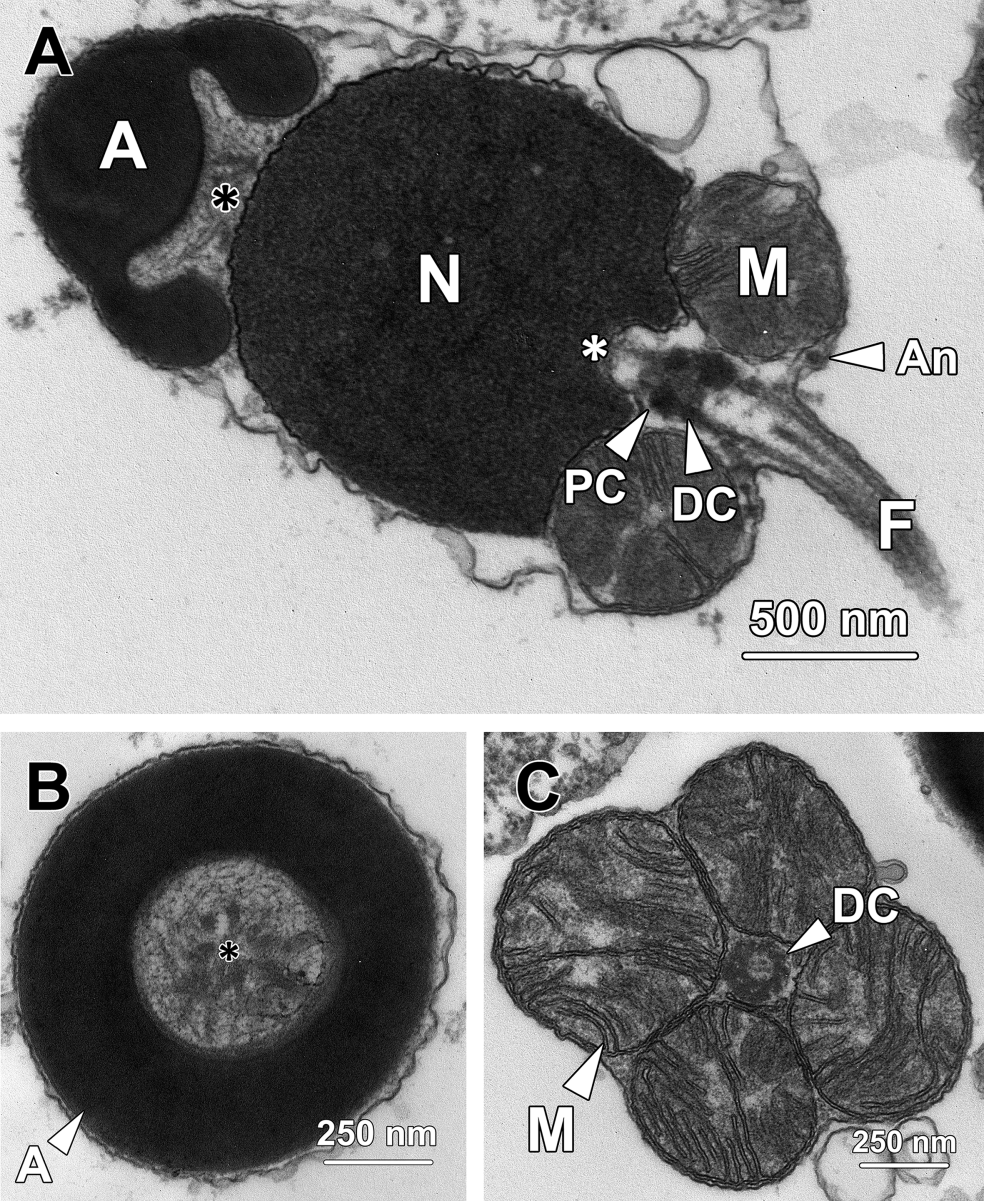
Transmission electron micrographs of spermatozoa. **(A)** Spermatozoa consist of a cap-like acrosome (A), an oval nucleus (N), a short midpiece containing four spherical mitochondria (M) and an elongated flagellum (F). A subacrosomal space (*, black asterisk) forms between the acrosome and the nucleus, containing evenly distributed electron-dense fibrillary subacrosomal material. The proximal centriole (PC) is inserted into the implantation fossa (*, white asterisk) formed at the posterior end of the nucleus. The four mitochondria lodge in concave recesses at the posterior nuclear margin and surround the base of the flagellum, where the annulus (An) forms. **(B)** A transverse section through the ring structure at the base of the acrosome shows the fibrillary content of the subacrosomal space (*, black asterisk). **(C)** A transverse section through the midpiece shows four spherical mitochondria (M) encircling the base of the flagellum.

From the head to the tail, the spermatozoa in *G*. *gemineoa* consisted of a cap-like acrosome (A), an oval nucleus (N) containing fully condensed homogeneous chromatin, a short midpiece containing four spherical mitochondria and an elongated flagellum (Fig 11). The acrosome exhibited a homogeneous appearance with evenly distributed electron density. The posterior end of the acrosome was in close proximity to but not directly attached to the nucleus, forming a subacrosomal space (*, black asterisk; Fig 11A) that contained electron-dense fibrillary material in a homogeneous granular subacrosomal matrix (Fig 11A and 11B). At the posterior end of the nucleus, the proximal centriole was inserted into the shallow implantation fossa (*, white asterisk; Fig 11A). The four spherical mitochondria firmly adhered to corresponding depressions of the nucleus and surrounded the base of the flagellum, where the annulus, an electron-dense ring-shaped structure formed (Fig 11A and 11C).

**Fig 12:**
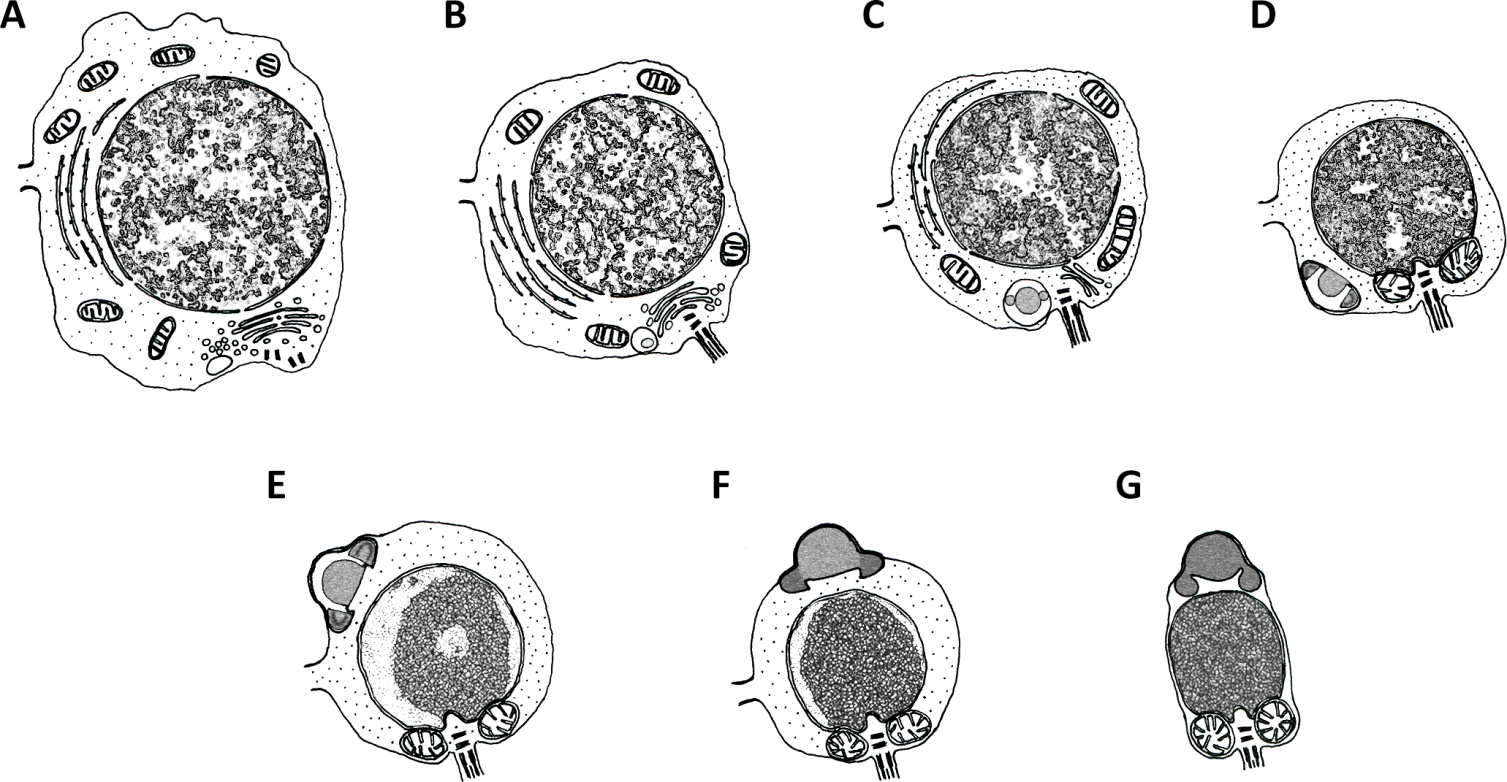
Illustrations showing the pattern of spermiogenesis in *G*. *gemineoa*. Spermiogenesis can be temporally divided into **(A—B)** Golgi phase, **(C—E)** acrosomal phase and **(F—G)** maturation phase. **(A)** In early Golgi phase, a large number of single-membrane bound vesicles are observed adjacent to a conspicuous Golgi complex. These vesicles gradually merge into an electron-lucent proacrosomal vacuole. A pair of centrioles, which were initially located in close proximity to the nuclear membrane, migrate towards the cell membrane. **(B)** In the late Golgi phase, the fusion of proacrosomal vesicles ceases and a patch of electron-dense material forms in the matrix of the proacrosomal vacuole. The distal centriole docks to the cell membrane, where a formative flagellum subsequently protrudes out. **(C)** In early acrosomal phase, the Golgi complex becomes no longer involved in the differentiation of the acrosome and the proacrosomal vacuole transforms into a proacrosome containing a large sphere surrounded by a ring-like structure. The chromosomes decondense into rod-shaped chromatin blocks and occupy half volume of the nucleoplasm. **(D)** The proacrosome transforms into an inverted-bowl shaped acrosome, during which both the central sphere and the ring structure attach to the inner posterior membrane of the acrosome. The proximal centriole comes into intimate contact with the implantation fossa formed at the posterior end of the nucleus. After the fusion of mitochondria, they migrate to and adhere to concave recesses of the nucleus at the posterior pole. The acrosome starts to migrate towards the anterior pole of the cytoplasm after the adherence of the mitochondria. **(E)** In late acrosomal phase, the acrosome almost reaches the most anterior aspect of the spermatids. The chromatin condenses into a rough oval-shaped chromatin block. **(F)** In the early maturation phase, the acrosome has accomplished the majority of its differentiation, during which the central hemisphere comes to occupy the entire matrix of the acrosome. **(G)** In the late maturation phase, acrosome differentiation is completed and the nucleus is fully condensed. The excess cytoplasm is completely eliminated from the spermatid through the extrusion of residual bodies. The cytoplasmic bridge between the spermatids ruptures and the spermatids develop into individual spermatozoa.

Fig 12 illustrated the pattern of spermatid differentiation in *G*. *gemineoa*. Temporally, the entire spermiogenesis could be divided into three phases: Golgi phase, acrosomal phase and maturation phase. The Golgi phase involved the formation and growth of proacrosomal vacuole, the migration of the centrioles and the formation of flagellum (Fig 12A to 12B). During the acrosomal phase, the acrosome accomplished a majority of its differentiation and the nucleus decreased in size in association with chromatin condensation (Fig 12C to 12E). Spermiogenesis in *G*. *gemineoa* was concluded during the maturation phase, during which the acrosome finalised its migration and differentiation, the nucleus accomplished its condensation and excess cytoplasm was extruded from the spermatids in the form of residual bodies (Fig 12F to 12G). With the elimination of residual bodies, the cytoplasmic bridge that connected the spermatids broke down and the spermatids became independent from each other and developed into spermatozoa (Fig 12G).

### Recapitulation of spermatid differentiation *in vitro*

**Fig 13:**
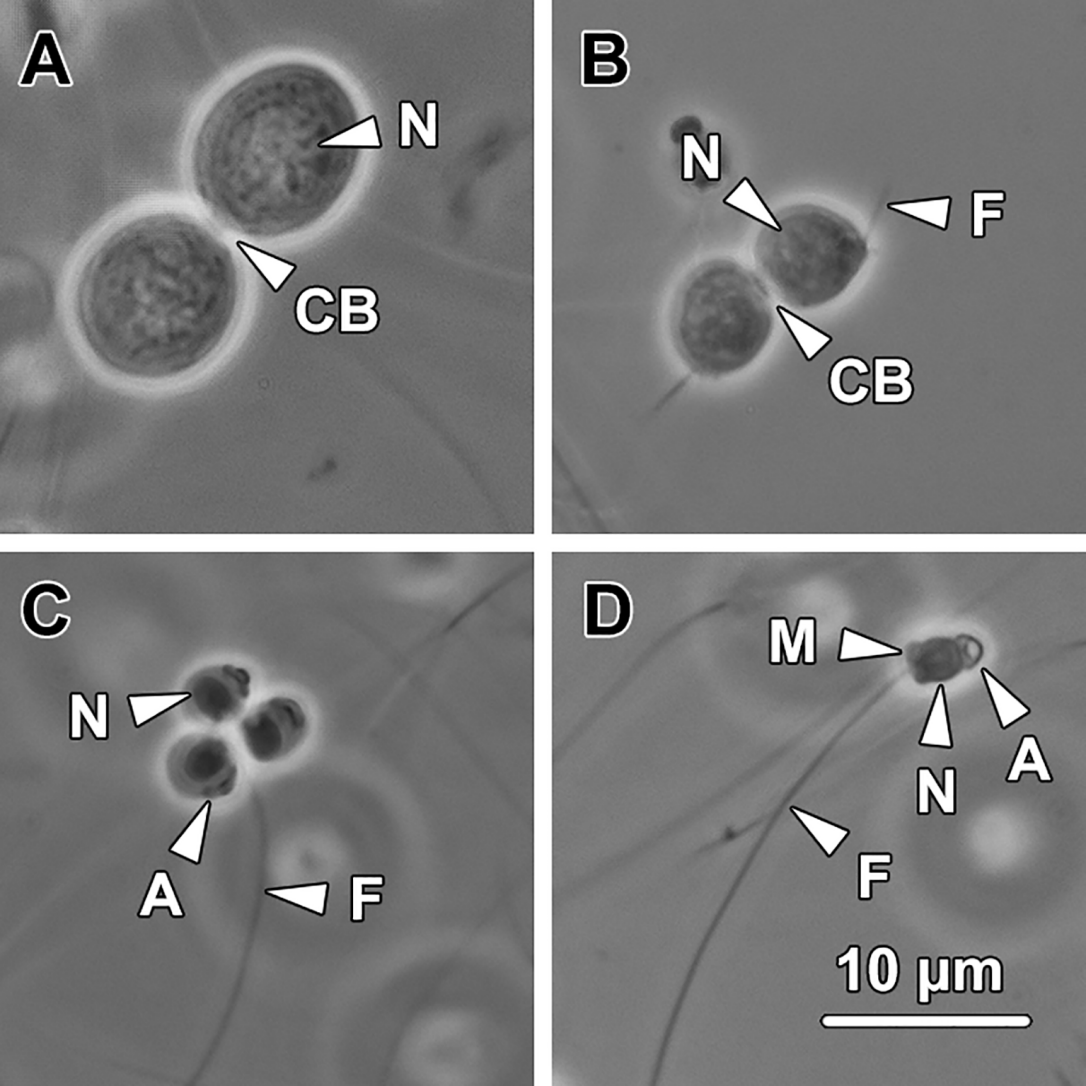
Representative types of spermatids and spermatozoa in the culture media. **(A)** Step 1–3 spermatids. The spermatids at these steps are spherical to oval in shape. They are in pairs and interconnected by a cytoplasmic bridge (CB). N, nucleus. **(B)** Step 4–6 spermatids. Spermatids at these stages remain connected by a cytoplasmic bridge (CB) however a short flagellum (F) protrudes from the posterior end of each spermatid. N, nucleus. **(C)** Step 7–9 spermatids. The spermatids at these steps are spherical and contain a cap-like acrosome (A), a condensed nucleus (N) and an elongated flagellum (F). The acrosome resides at or near the posterior end of the spermatids. **(D)** Spermatozoa. Spermatozoa consist of a cap-like acrosome (A), an oval nucleus (N), a short midpiece (M) and a flagellum (F).

After being purified through discontinued Percoll gradients, three major types of spermatids were commonly found in the culture media: spherical- to oval-shaped spermatids with no flagellum (step 1–3 spermatids; Fig 13A); spherical-shaped spermatids with short flagella (step 4–6 spermatids; Fig 13B); spherical-shaped spermatids with conspicuous acrosome, condensed nucleus and elongated flagellum (step 7–9 spermatids; Fig 13C). In addition, a small number of spermatozoa remain in the culture medium (Fig 13D). The percentage of each type of differentiating spermatid was recorded at 0 h, 12 h, 24 h and 36 h respectively after incubation. These data were used to determine whether the applied culture medium was effective in inducing spermiogenesis *in vitro*, or not.

**Fig 14:**
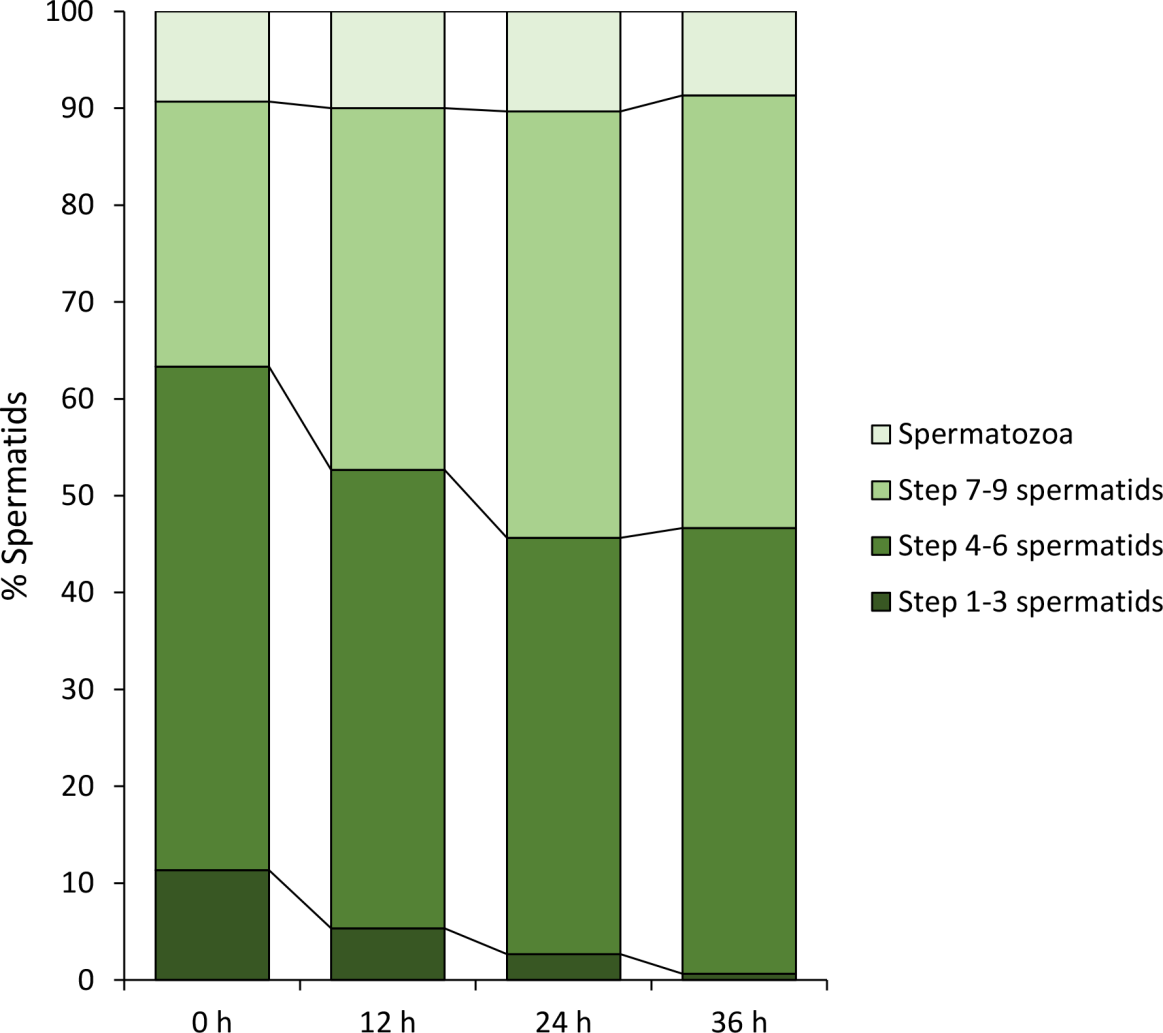
The male germinal fluid is able to induce a substantial portion of spermatid differentiation *in vitro*. After 36 h of incubation in the male germinal fluid, the average percentage of step 1–3 spermatids decreases from 11.3% to 0.7%, whereas that of step 7–9 increases from 27.3% to 44.7%. The percentages of step 4–6 slightly reduce from 52% to 47.3% in the first 12 h and become constant afterwards. The percentage of spermatozoa remains almost unchanged (~ 10%) during the entire 36 h-incubation, indicating no new spermatozoon has generated in this culture media.

Since spermiogenesis in *G*. *gemineoa* occurs while floating freely in the germinal fluid, we first attempted to induce the differentiation of spermatids *in vitro* by using germinal fluid obtained from male adults. Within 36 h of incubation, the percentage of step 1–3 spermatids dramatically decreased from 11.3% to 0.7% (P < 0.01) while the average percentage of step 7–9 spermatids increased from 27.3% to 44% during the first 24 h and remained constant during the last 12 h of incubation (Fig 14). The average percentage of step 4–6 spermatids slightly decreased from 52% to 47.3% in the first 12 h and maintained at around 45% during the remaining 24 h of incubation (Fig 14). The ratios of these three types of spermatids at the four time points indicated that the male germinal fluid was able to support the differentiation of step 1–3 spermatids to step 7–9 spermatids within 36 h. However, the percentage of spermatozoa remained nearly the same (~ 10%) during the entire 36 h-incubation (Fig 14), suggesting the male germinal fluid under current culture conditions was unable to support the differentiation of step 7–9 spermatids into spermatozoa.

**Fig 15:**
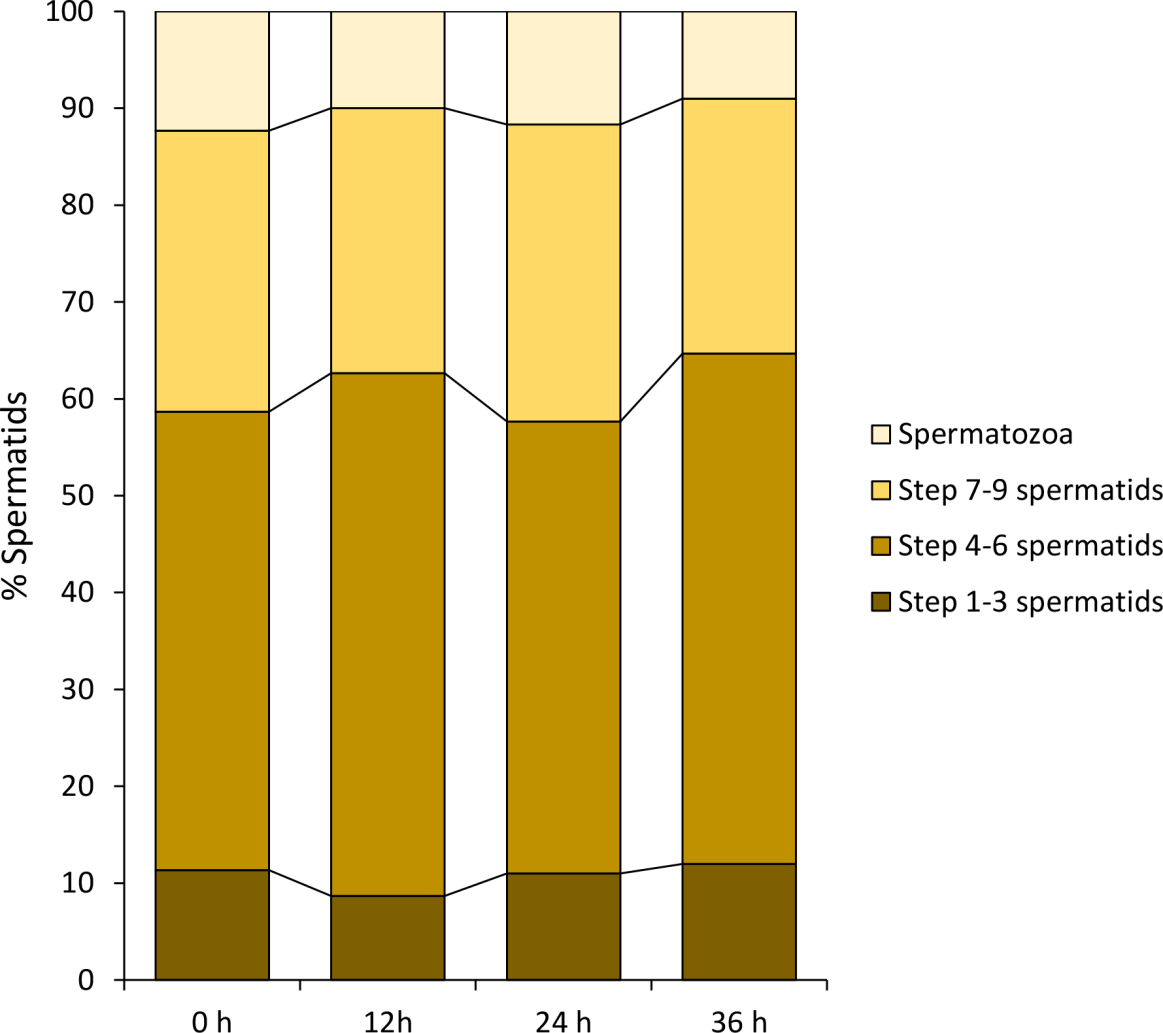
The boiled male germinal fluid loses its capacity to support the differentiation of spermatids *in vitro*. During the 36 h of incubation in the boiled male germinal fluid, no significant change of the average percentages is detected in all four types of sperm cells (P > 0.05). It indicates that the boiled male germinal fluid cannot induce the young spermatids to differentiate *in vitro*, but can maintain the viability of the spermatids for at least 36 h.

To determine whether any protein regulator of spermiogenesis existed in the male germinal fluid, the spermatids were cultivated in boiled male germinal fluid under the same culture conditions. Throughout the 36 h of incubation, the average percentages of all four types of sperm cells were generally unchanged (P > 0.05; Fig 15). As the boiled male germinal fluid failed to support the differentiation of spermatids *in vitro*, the regulators of spermiogenesis in the male germinal fluid of *G*. *gemineoa* most likely contained molecules, such as proteins, that were thermolabile. Since no significant degeneration of spermatids occurred during the 36 h of incubation in the boiled male germinal fluid, it is also suggested that this culture medium was able to maintain the viability of spermatids for at least 36 h–but not their differentiation.

**Fig 16:**
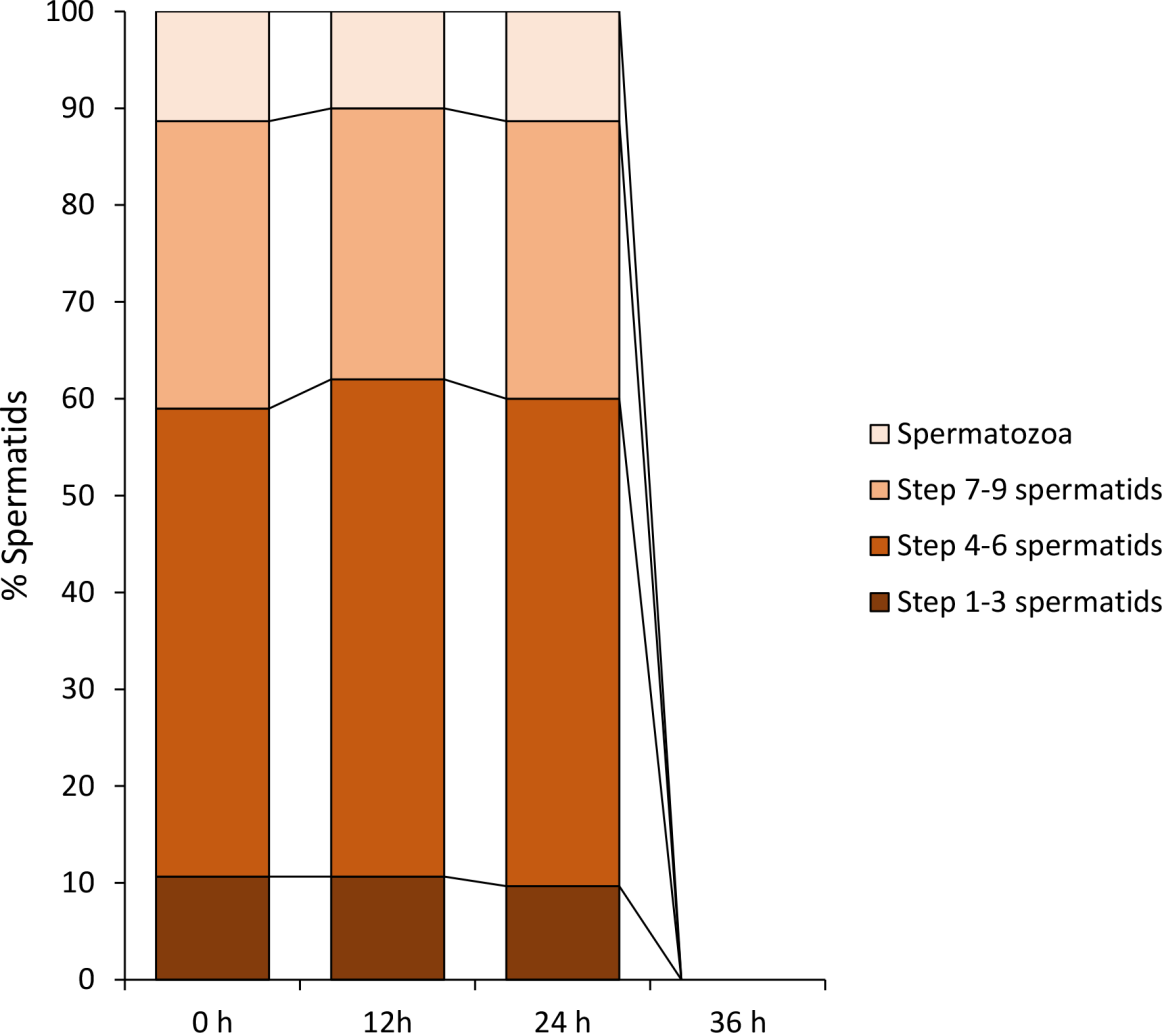
The female germinal fluid is unable to induce the differentiation of spermatids *in vitro*. During the first 24 h of incubation, the percentages of step 1–3 spermatids, step 7–9 spermatids and spermatozoa are almost unchanged (P > 0.05). The spermatids degenerated after 24 h-incubation in the female germinal fluid; therefore, data are not obtainable at 36 h.

To determine whether the regulators of spermiogenesis were gender-specific, spermatids were then incubated in female germinal fluid under the same culture conditions. Within 24 h of incubation, the percentages of the four types of sperm cells exhibited no significant change (P > 0.05; Fig 16), suggesting that female germinal fluid could not support the differentiation of spermatids. The spermatids cultured in the female germinal fluid started to degenerate within 24 h after incubation, making it impossible to obtain any data at 36 h of incubation.

**Fig 17:**
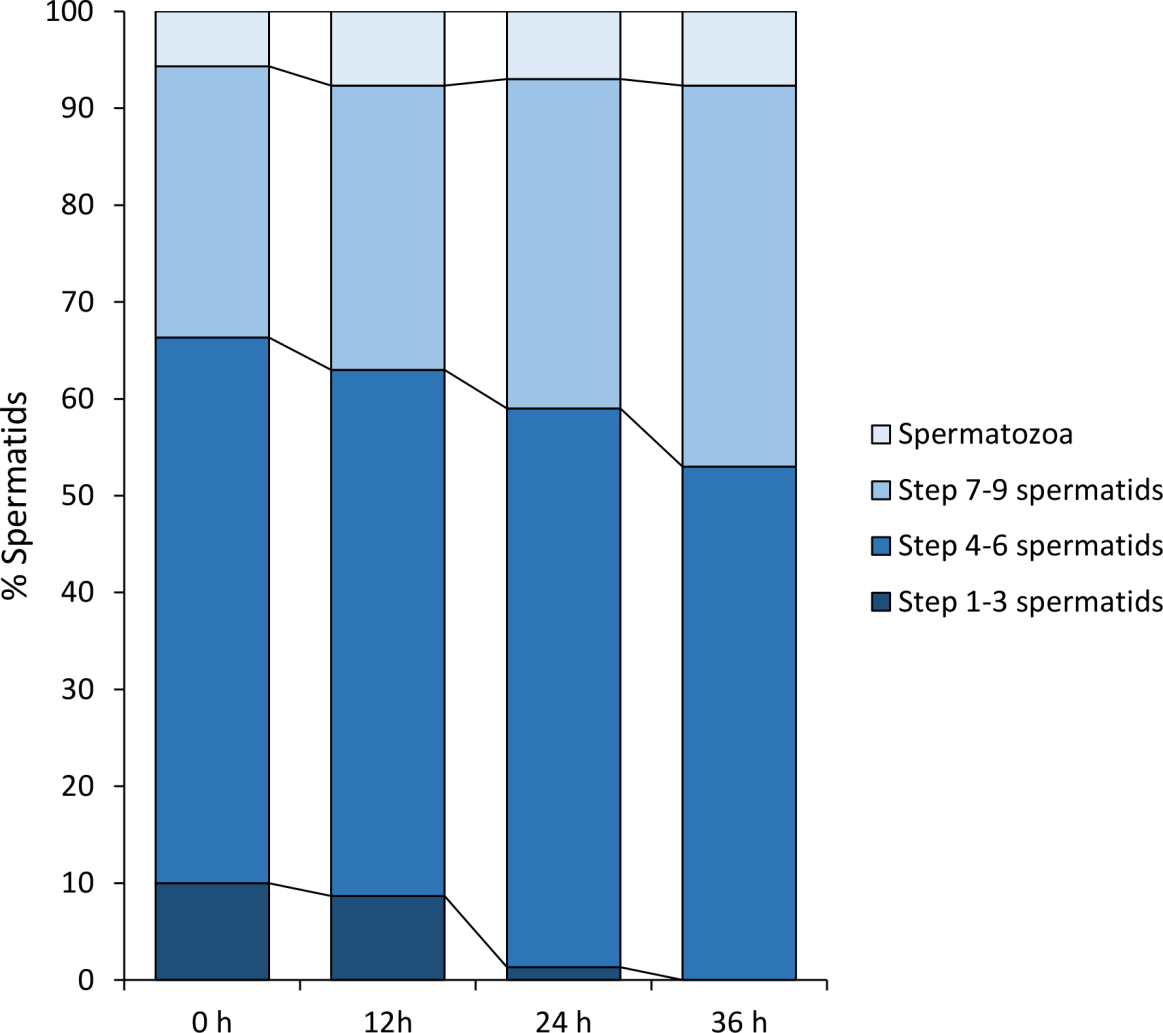
10% foetal bovine serum in RPMI 1640 medium is able to induce a substantial portion of spermatid differentiation *in vitro*. After 36 h of incubation in this culture medium, the percentage of step 1–3 spermatids decreases from 10% to 0% while that of step 7–9 spermatids increases from 28% to 39.3%. The average percentages of step 4–5 spermatids and spermatozoa exhibit no significant change, which remain about 55% and 7% respectively (P > 0.05).

The spermatids were finally incubated in 10% foetal bovine serum in RPMI 1640 medium (RPMI + FBS) under the same *in vitro* culture conditions. Within 36 h of incubation, the percentage of step 1–3 spermatids reduced from 10% to 0% (P < 0.01; Fig 17) while the percentage of step 7–9 spermatids increased from 28% to 39.3% (P < 0.01; Fig 17). The percentages of step 4–6 spermatids and spermatozoa were almost constant, which were about 55% and 7% respectively (P > 0.05; Fig 17).

## Discussion

Spermiogenesis in mammals generally occurs within the seminiferous epithelium and is completely dependent upon the adherent Sertoli cells. These nurse cells provide the differentiating sperm cells with essential nourishment, such as growth and anti-apoptotic factors and form the blood-testis barrier to support the movement of these sperm cells towards the lumen of seminiferous tubule during the differentiation process. Such dependence upon supporting cells renders mammalian spermiogenesis extremely difficult to replicate in completely artificial environments. On the contrary, spermiogenesis in *G*. *gemineoa* takes place while the spermatids are floating freely in the germinal fluid. This species may therefore make a convenient model to study germ cell differentiation *in vitro* with the intention of unlocking the molecular mechanisms that underpin this process. However, the steps of spermatid differentiation in polychaetes are particularly hard to identify, as the spermatids exhibit similar size and appearance to their spermatogenic precursors and are randomly dispersed in the lumen of the germinal chamber. Given these factors, spermiogenesis in a large proportion of polychaetes has not been adequately described.

In *G*. *gemineoa*, the acrosome first appeared as a cluster of single membrane-bound proacrosomal vesicles situated at the posterior pole of the spermatids and in close apposition to the concave surface of the Golgi complex. The involvement of Golgi complex in forming the proacrosome is probably a ubiquitous feature of acrosome development as demonstrated in many other polychaetes (e.g. *Phragmatopoma lapidosa* [21] and *Capitella* spp. [22]), and avian (e.g. *Coturnix coturnix japonica* [23]) and mammalian species (e.g. *Ornithorhynchus anatinus* [24]). In these species, including *G*. *gemineoa*, the proacrosomal vacuole forms similarly through the fusion of the proacrosomal vesicles. The proacrosomal vacuole in *Ornithorhynchus anatinus* [24] approaches the nuclear envelope and lodges into a concavity formed at the anterior pole of the nucleus. After the lodgement of the proacrosome, it transforms into a cap-shaped structure covering about two thirds of the nuclear surface; this process is known as the cap phase of spermiogenesis in mammals. In contrast, the cap phase does not exist in the spermiogenesis of *G*. *gemineoa* and other polychaetes that have been studied (e.g. *Phragmatopoma* lapidosa (Sabellariidae) [21] and *Vanadis formosa* (Alciopidae) and *Krohnia lepidota* (Alciopidae) [25]). The acrosome in these polychaetes is relatively free and never attaches to the nuclear membrane during the entire spermiogenic process. During the Golgi phase of spermiogenesis in *G*. *gemineoa*, a pair of centrioles was first found residing adjacent to both the nuclear membrane and the Golgi complex, which is consistent with that in the step 1 spermatid of the mammal *Ornithorhynchus anatinus* [24]. In both species, the two centrioles gradually migrate towards the plasma membrane and the distal centriole becomes docked with the cell membrane, where it subsequently gives rise to a flagellum, suggesting the convergence of developmental mechanisms around a common functional need–the creation of a highly motile male gamete.

During the early acrosomal phase, an implantation fossa was formed at the posterior pole of the nucleus, followed by the insertion of the proximal centriole. The implantation fossa in *G*. *gemineoa* is relatively shallow compared with that in *Marenzelleria viridis* [26], the depth of whose implantation fossa accounts for approximately half the length of the longitudinal axis of the nucleus. However, the proximal centriole in *G*. *gemineoa* made contact with the fossa by extending its microtubules into it, whereas in all mammalian species examined this centriole directly attaches to the implantation fossa.

After insertion of the proximal centriole into the implantation fossa, the mitochondrial sheath started to form, as indicated by changes in the number, size and position of the mitochondria. From the Golgi phase to the early acrosomal phase, small spherical mitochondria, which were initially randomly dispersed in the cytoplasm, fused with each other to form larger spherical-shaped mitochondria that migrated towards the posterior end of the spermatids. Fusion of mitochondria has been recognised as a common process during spermiogenesis in all polychaetes studied and is necessary for the reduction of mitochondrial number and the subsequent formation of the mitochondrial sheath [27]. During the step 3 of spermiogenesis in *G*. *gemineoa*, larger rod-shaped mitochondria, which were twice as large as the small spherical mitochondria started to appear, indicating the occurrence of mitochondrial fusion in the spermatids. Interestingly, the fused mitochondria in *G*. *gemineoa* became lodged into depressions formed at the posterior end of the nucleus. Such adherence of mitochondria to concave recesses in the envelope has also been found in ect-aquasperm (e.g. *Lepidonotus* sp. [17], *Marenzelleria viridis* [26], *Phragmatopoma lapidosa* [21], *Serpula* sp., *Streblosoma acymatum* and *Tylorrhynchus heterochaetus* [17]) and ent-aquasperm (e.g. *Clymenella* sp. [17]) with a short midpiece. This feature, however, cannot be observed in ent-aquasperm (e.g. *Fabricia* sp., *Oriopsis* sp. and *Chitinopoma serrula* [17]) and introsperm (e.g. *Questa* sp. and *Tripolydora* sp. [17]) with an elongated midpiece. During the late acrosomal phase of spermatogenesis in *G*. *gemineoa*, the fused mitochondria formed the mitochondrial sheath surrounding the principal segment of the flagellum. Such an intimate relationship between the centrioles and mitochondria may permit fast diffusion of ATP, which is essential for sperm movement [22]. Comparatively, in *Ornithorhynchus anatinus* [24] and many other mammalian species, the spermatozoa contain a large number of mitochondria (50–75 rather than the 4 in *G*. *gemineoa*), generating a relatively long midpiece at the junction of the nucleus and the flagellum.

In *Ornithorhynchus anatinus*, both the acrosome and flagellum initially appeared at the anterior end of the cytoplasm and the flagellum subsequently migrated towards the opposite end where it became associated with the implantation fossa [24]. However, in *G*. *gemineoa*, the flagellum remained located at the posterior end of the spermatids, where the acrosome first appeared and the mitochondrial sheath later formed. Moreover, instead of the flagellum migrating to the pole opposite the acrosome, the acrosome in the spermatids of *G*. *gemineoa* was the one that migrated. Acrosome migration during spermiogenesis has also been discovered in other polychaetes, such as *Marenzelleria viridis* [16], *Phragmatopoma lapidosa* [21] and *Capitella* spp. [22]. In these species, including *G*. *gemineoa*, the acrosome forms and migrates from the posterior end of the cytoplasm to the anterior end. The migration generally initiates on completion of nuclear condensation and is accomplished before the final differentiation of the acrosome. Migration of the acrosome in *G*. *gemineoa* happens while it is in intimate association with the cytoplasmic membrane [28]. By contrast, in *Ornithorhynchus anatinus* and most other mammalian species, after the lodgement in a concave recess at the anterior aspect of the nucleus, the acrosome persists adhering to the nuclear membrane throughout the entire spermiogenic process [24], highlighting a potential inter-species differences in the mechanisms regulating acrosomal migration.

Nuclear condensation was generally accomplished during the acrosomal phase of spermiogenesis in *G*. *gemineoa*. Microtubules were found running around the nucleus in the spermatids of *G*. *gemineoa* and *Streblospio benedicti* [26]. The bundles of microtubules, or the manchette, have been demonstrated to play a role in shaping the nucleus in polychaetes (e.g. *Krohnia lepidota* [25] and *Capitella* spp. [22]). Instead of occurring simultaneously, the compaction of the chromatin in the spermatids of *G*. *gemineoa* occurs prior to the shrinkage of the nuclear envelope. The heterochromatin first aggregates into an irregular-shaped chromatin block attaching to the posterior end of the inner nuclear membrane, where the implantation fossa formed. Such chromatin aggregation at the posterior nucleus has also been observed in older spermatids of *Phragmatopoma lapidosa* [21]. In *G*. *gemineoa*, after adherence of the spherical mitochondria to the nuclear envelope, dramatic chromatin aggregation and shaping occurred. Franzén and Rice [28] suggested that the chromatin condensation during this period might be mediated by these mitochondria associated with the nuclear membrane. In polychaete species such as *Polydora* spp. [29] and *Streblospio benedicti* [26], chromatin condensation involves heterochromatin transforming from a fibrillary or lamellar structure into homogeneously electron-dense material during late spermiogenesis. However, the homogeneous chromatin in spermatozoa is transformed from granular material in several other polychaetes, such as in *Amathys lutzi* [30], *Aonides oxycephala* [31] and *Terebrasabella heterouncinata* [32]. In *G*. *gemineoa*, the fully condensed chromatin in the sperm nucleus retained a densely packed granular texture, as has been observed in other polychaete species *Sabella spallanzanii* [33], *Gorgoniapolynoe caeciliae* [34] and *Methanoaricia dendrobranchiata* [15] reflecting the dense granular chromatin material typical of mammalian spermatozoa.

In *G*. *gemineoa*, the subacrosomal space was not filled with granular subacrosomal material until the lower end of the ring-like structure closely approached the apex of the nucleus. Sparsely distributed fibrillary material was observed along the base of the subacrosomal space in *G*. *gemineoa* and in some other polychaetes, such as *Phragmatopoma lapidosa* [21] and *S*. *tetraceros* [35]. The electron-dense fibrillary material, known as subacrosomal filaments, are presumed to be actin, which plays an important role in the acrosome reaction during fertilisation [21]. The acrosome in the spermatozoa of *G*. *gemineoa* exhibited a cap-like appearance, which is similar to the acrosomal morphology observed in several other closely related polychaete species with ect-aquasperm (e.g. *Hydroides dianthus*, *Serpula vermicularis*, *Vermiliopsis infundibulum* [36] and *Spirobranchus tetraceros* [35]).

The *in vitro* study in this article demonstrated that step 1–3 spermatids were able to differentiate into step 7–9 spermatids in both male germinal fluid (a natural culture medium) and 10% foetal bovine serum (FBS) in RPMI 1640 medium (an artificial culture medium). Spermatid differentiation failed to recapitulate in female or boiled male germinal fluids, suggesting the essential regulators of spermiogenesis in *G*. *gemineoa* were male-specific and contained key proteins. *In vitro* recapitulating the complex differentiation of male germ cells from diploid spermatogonial stem cells to highly specialised haploid spermatozoa has been a quest of great interest over the last century [37, 38]. The establishment of an *in vitro* culture system capable of producing functional spermatozoa is expected to facilitate mechanistic studies of spermatogenesis, directed towards genetic manipulation of the male germ line and treatment of male factor infertility [39].

To date, the recapitulation of spermatogenesis *in vitro* has been achieved in several types of fish, including Japanese eel (*Anguilla japonica* [40]), zebrafish (*Danio rerio* [41]) and medaka (*Oryzias latipes* [42, 43]) suggesting that this aim is potentially achievable. Spermatogenesis in Japanese eel was reproduced *in vitro* using an organ culture system. Immature eel testes, containing premitotic spermatogonia, type A and early type B spermatogonia only, were isolated and incubated for 21 d to harvest functional spermatozoa [40]. During this study, Miura et al. found that the addition of 11-ketotestosterone, a potent androgen in teleosts, to the culture medium was critical for the success of the *in vitro* culture. On the basis of such findings, they deduced that hormonal induction of spermatogenesis in the Japanese eel involved gonadotrophin stimulation of Leydig cells to produce 11-ketotestosterone [40]. This steroid hormone, in turn, activates Sertoli cells to stimulate spermatogonial stem cells to initiate mitosis and complete spermatogenesis [44]. Spermatogenesis in zebrafish has been recapitulated *in vitro* by co-culturing the isolated testicular cells containing germ cells with feeder cells (i.e. epithelial and fibroblast cells) derived from tumour-like hypertrophied testis. These feeder cells were found to express the Sertoli-specific phagocytic potential and were demonstrated to stimulate proliferation of spermatogonial stem cells [41]. The success of such two-cell culture systems was heavily dependent on either the original testicular supporting cells or cells capable of mimicking Sertoli cell functions. Distinctly, the *in vitro* recapitulation of spermatogenesis in medaka fish, as in the present study, was achieved by culturing isolated germ cells in a completely artificial culture medium. Functional spermatozoa were produced after 10 d-incubation of spermatocytes in Leibovitz-15 medium containing 10% FBS [43]. Under *in vivo* conditions, spermatogenesis in this teleost has been demonstrated to depend on a monolayer of lobule boundary cells, recognised as homologues of Sertoli cells in mammals. These supporting cells were found to ingest the residual cytoplasm released by older spermatids [45]. Interestingly, the shedding of residual bodies has also been observed *in vitro* in teleosts, without any assistance from supporting cells. In this regard, Saiki et al. [43] believed that in lower order vertebrates, although the progression of spermatogenesis was controlled by testicular somatic cells, the presence of these cells might not be necessary for the *in vitro* differentiation of spermatocytes to spermatids.

In mammalian species, *in vitro* recapitulation of spermatogenesis has remained elusive until Sato et al. [46] successfully produced fully functional spermatozoa from neonatal mouse testes containing primordial germ cells. In this study, the researchers discovered that FBS was indispensable to induce the differentiation of spermatogonia to haploid round spermatids, but using this medium, these authors were unable to produce elongating spermatids or spermatozoa. Such results raised the possibility that FBS contained substances capable of suppressing further progress of spermatogenesis. By replacing FBS with FBS-free KnockOut Serum Replacement (KSR; Invitrogen) medium, Sato et al. achieved success in producing functional spermatozoa utilising their culture system. They suggested that further identification of key molecules in KSR medium was necessary for uncovering the mechanisms of spermatogenesis and might facilitate further optimisation of their cell culture conditions [46].

Since the current *in vitro* culture system employing *G*. *gemineoa* gametes was unable to support the differentiation of young spermatids into functional spermatozoa, it is clear that refinement of the culture system for this species is necessary. On the basis of the aforementioned studies that successfully recapitulated spermatogenesis *in vitro*, the refinement could be implemented following three aspects: (1) hormones might be involved in the regulation of spermatogenesis in polychaetes. By conducting analyses of endocrine activity during spermatogenesis in *Nereis diversicolor*, Bertout [47] discovered that spermatogenesis in this polychaete species was regulated by an undefined hormone secreted by its brain. This hormone was demonstrated to inhibit the differentiation of spermatocytes and removal of this hormone would lead to increased RNA synthesis, followed by DNA replication and meiotic divisions. Therefore, studies should be undertaken to investigate if key hormones are involved in the regulation of spermatogenesis in *G*. *gemineoa*. (2) A prolonged culture system should be maintained, as it might take days to weeks to produce functional spermatozoa *in vitro*. In the current study, young spermatids in *G*. *gemineoa* were stimulated to differentiate into older ones in a closed culture system, in which supplemental nutrient was not added and waste products were not removed. Therefore, an open culture system with well-maintained culture conditions (e.g. illumination, humidity, pH and salinity) should be established. (3) Multiple culture media might be utilised to meet the requirement of germ cells at different spermatogenic stages. As indicated by Hunter et al. [38], spermatogenic cells at different developmental stages appeared to have different culture requirements, in other words, a single culture medium might not be able to recapitulate the entire process of spermatogenesis. For example, the study conducted by Sato et al. [46] demonstrated that FBS was only able to support the differentiation of spermatogonial stem cells into round spermatids and the presence of FBS suppressed the germ cells from differentiating into elongating spermatids and functional spermatozoa.

As a platform for the use of *G*. *gemineoa* as a bio-indicator species capable of detecting the presence of pollutants targeting male reproduction, we have described, for the first time, the details of male germ cell differentiation in this species. While certain features of sperm production in this species are phylogenetically determined (e.g. the morphogenesis of the acrosome or the number and positioning of midpiece mitochondria), the functional constraints imposed by fertilisation also lead to similarities across species in the organisation, ontogeny and operation of male gametes. Thus, such general features of sperm production in *G*. *gemineoa* as the development and functional organisation of the sperm tail, the location of proximal and distal centrioles in the sperm neck, cytoplasmic reduction and chromatin condensation, support the potential value of species as a model organism capable of responding to environmental stress and generating data of relevance across species boundaries. Future studies might, for example, explore this bio-indicator role using chemicals that we know are present in the biosphere and are thought to impact on human reproductive competence such as bisphenol A or phthalate esters. If this species is to be used as a model for studying the mechanisms by which environmental pollutants exert their clinical effects, it would be of immense value if we could recapitulate the spermatogenic process *in vitro*. Although the *in vitro* culture system described in this study was very preliminary, it has provided direction for further investigation on the mechanisms of spermatogenesis in *G*. *gemineoa* and supported our concept that spermiogenesis in this species was highly dependent on intrinsic programming in the germ line. Given the recent publication of a meta-regression analysis reporting a 50% decline in human sperm counts over the past half century, the development of animal models that can be used to reflect the presence of environmental toxicants capable of disrupting male reproduction is particularly significant [48].

## References

[1] Rouse GW. The Annelida In: Anderson DT, editor. Invertebrate zoology. South Melbourne: Oxford University Press; 1998 pp. 196–200.

[2] Hutchings P. Biodiversity and functioning of polychaetes in benthic sediments. Biodivers Conserv. 1998;7(9): 1133–1145. doi: 10.1023/A:1008871430178

[3] Edgar GJ. Australian marine life: the plants and animals of temperate waters. Sydney: New Holland; 2008.

[4] Styan CA, Kupriyanova EK, Havenhand JN. Barriers to cross-fertilization between populations of a widely dispersed polychaete species are unlikely to have arisen through gametic compatibility arms-races. Evolution. 2008;62(12): 3041–3055. doi: 10.1111/j.1558-5646.2008.00521.x 18803690 10.1111/j.1558-5646.2008.00521.x

[5] Halt MN, Kupriyanova EK, Cooper SJ, Rouse GW. Naming species with no morphological indicators: species status of *Galeolaria caespitosa* (Annelida: Serpulidae) inferred from nuclear and mitochondrial gene sequences and morphology. Invertebr Syst. 2009;23(3): 205–222. doi: 10.1071/IS09003

[6] Bremner J. Species' traits and ecological functioning in marine conservation and management. J Exp Mar Biol Ecol. 2008;366(1–2): 37–47. doi: 10.1016/j.jembe.2008.07.007

[7] Dean HK. The use of polychaetes (Annelida) as indicator species of marine pollution: a review. Rev Biol Trop. 2008;56(4): 11–38.

[8] Waring JS, Maher WA, Krikowa F. Trace metal bioaccumulation in eight common coastal Australian polychaeta. J Environ Monit. 2006;8(11): 1149–1157. doi: 10.1039/b612509n 17075622 10.1039/b612509n

[9] Andrews J, Anderson D. The development of the polychaete *Galeolaria caespitosa* Lamarck (Fam. Serpulidae). Proc Linn Soc N S W. 1962;87(2): 185–188.

[10] Kupriyanova EK. Fertilization success in *Galeolaria caespitosa* (Polychaeta: Serpulidae): gamete characteristics, role of sperm dilution, gamete age, and contact time. Sci Mar. 2006;70(S3): 309–317. doi: 10.3989/scimar.2006.70s3309

[11] Marshall D, Evans J. Does egg competition occur in marine broadcast-spawners? J Evol Biol. 2005;18(5): 1244–1252. doi: 10.1111/j.1420-9101.2005.00947.x 16135120 10.1111/j.1420-9101.2005.00947.x

[12] Marshall D, Evans J. The benefits of polyandry in the free-spawning polychaete *Galeolaria caespitosa*. J Evol Biol. 2005;18(3): 735–741. doi: 10.1111/j.1420-9101.2004.00873.x 15842502 10.1111/j.1420-9101.2004.00873.x

[13] Falkenberg LJ, Havenhand JN, Styan CA. Sperm Accumulated Against Surface: A novel alternative bioassay for environmental monitoring. Mar Environ Res. 2016;114: 51–57. doi: 10.1016/j.marenvres.2015.12.005 26763685 10.1016/j.marenvres.2015.12.005

[14] Schlegel P, Havenhand JN, Obadia N, Williamson JE. Sperm swimming in the polychaete *Galeolaria caespitosa* shows substantial inter-individual variability in response to future ocean acidification. Mar Pollut Bull. 2014;78(1–2): 213–217. doi: 10.1016/j.marpolbul.2013.10.040 24239098 10.1016/j.marpolbul.2013.10.040

[15] Eckelbarger KJ, Young CM. Spermiogenesis and modified sperm morphology in the “seepworm” *Methanoaricia dendrobranchiata* (Polychaeta: Orbiniidae) from a methane seep environment in the Gulf of Mexico: implications for fertilization biology. Biol Bull. 2002;203(2): 134–143. doi: 10.2307/1543382 12414563 10.2307/1543382

[16] Bochert R. An electron microscopic study of spermatogenesis in *Marenzelleria viridis* (Verrill, 1873) (Polychaeta: Spionidae). Acta Zool. 1996;77(3): 191–199. doi: 10.1111/j.1463-6395.1996.tb01263.x

[17] Jamieson BGM, Rouse GW. The spermatozoa of the Polychaeta (Annelida): An ultrastructural review. Biol Rev. 1989;64(2): 93–157. doi: 10.1111/j.1469-185X.1989.tb00673.x 2675996 10.1111/j.1469-185x.1989.tb00673.x

[18] Rouse GW. Annelid sperm and fertilization biology. Hydrobiologia. 2005;535(1): 167–178.

[19] Rouse GW. Polychaete sperm: phylogenetic and functional considerations. Hydrobiologia. 1999;402(0): 215–224. doi: 10.1023/a:1003700827759

[20] Lu Y, Aitken RJ, Lin M. Detailed analysis of the male reproductive system in a potential bio-indicator species—the marine invertebrate *Galeolaria caespitosa* (Polychaeta: Serpulidae). PLoS ONE. 2017;12(4): e0174907 doi: 10.1371/journal.pone.0174907 28369153 10.1371/journal.pone.0174907 PMC5378414

[21] Eckelbarger KJ. Ultrastructure of spermatogenesis in the reef-building polychaete *Phragmatopoma lapidosa* (Sabellariidae) with special reference to acrosome morphogenesis. J Ultrastruct Res. 1984;89(2): 146–164. doi: 10.1016/S0022-5320(84)80011-8

[22] Eckelbarger KJ, Grassle JP. Spermatogenesis, sperm storage and comparative sperm morphology in nine species of *Capitella*, *Capitomastus* and *Capitellides* (Polychaeta: Capitellidae). Mar Biol. 1987;95(3): 415–429. doi: 10.1007/bf00409572

[23] Lin M, Jones R. Spermiogenesis and spermiation in the Japanese quail (*Coturnix coturnix japonica*). J Anat. 1993;183: 525–535. 8300429 PMC1259878

[24] Lin M, Jones R. Spermiogenesis and spermiation in a monotreme mammal, the platypus, *Ornithorhynchus anatinus*. J Anat. 2000;196(2): 217–232. doi: 10.1046/j.1469-7580.2000.19620217.x 10739018 10.1046/j.1469-7580.2000.19620217.x PMC1468055

[25] Rice SA, Eckelbarger KJ. An ultrastructural investigation of spermatogenesis in the holopelagic polychaetes *Vanadis formosa* and *Krohnia lepidota* (Polychaeta: Alciopidae). Biol Bull. 1989;176(2): 123–134. doi: 10.2307/1541579

[26] Rice SA. Spermatogenesis and sperm ultrastructure in three species of *Polydora* and in *Streblospio benedicti* (Polychaeta: Spionidae). Zoomorphology. 1981;97(1–2): 1–16. doi: 10.1007/BF00310099

[27] Purschke G, Fursman MC. Spermatogenesis and spermatozoa in *Stygocapitella subterranea* (Annelida, Parergodrilidae), an enigmatic supralittoral polychaete. Zoomorphology. 2005;124(3): 137–148. doi: 10.1007/s00435-005-0001-x

[28] Franzén Å, Rice SA. Spermatogenesis, male gametes and gamete interactions. The ultrastructure of polychaeta Microfauna Mar. 1988;4: 309–333.

[29] Gao Y, Zhang T, Zhang L, Qiu T, Xue D, Yang H. Ultrastructure developments during spermiogenesis in *Polydora ciliata* (Annelida: Spionidae), a parasite of mollusca. Journal of Ocean University of China. 2014;13(6): 1071–1077.

[30] Blake EA, Van Dover CL. The reproductive biology of *Amathys lutzi*, an ampharetid polychaete from hydrothermal vents on the Mid-Atlantic Ridge. Invertebr Biol. 2005;124(3): 254–264. doi: 10.1111/j.1744-7410.2005.00022.x

[31] Radashevsky VI, Alexandrova YN, Yurchenko OV. Spermiogenesis and spermatozoa ultrastructure of *Aonides oxycephala* (Annelida: Spionidae) from the Sea of Japan. Invertebrate Reproduction & Development. 2011;55(3): 168–174.

[32] Simon CA, Rouse GW. Ultrastructure of spermiogenesis, sperm, and the spermatheca in *Terebrasabella heterouncinata* (Polychaeta: Sabellidae: Sabellinae). Invertebrate Biology. 2005;124(1): 39–49.

[33] Giangrande A, Licciano M, Pagliara P, Gambi M. Gametogenesis and larval development in *Sabella spallanzanii* (Polychaeta: Sabellidae) from the Mediterranean Sea. Mar Biol. 2000;136(5): 847–861. doi: 10.1007/s002279900251

[34] Eckelbarger K, Watling L, Fournier H. Reproductive biology of the deep-sea polychaete *Gorgoniapolynoe caeciliae* (Polynoidae), a commensal species associated with octocorals. J Mar Biol Assoc U K. 2005;85(06): 1425–1433. doi: 10.1017/S0025315405012609

[35] Selim S, Naby FA, Gab-Alla A, Ghobashy A. Gametogenesis and spawning of *Spirobranchus tetraceros* (Polychaeta, Serpulidae) in Abu Kir Bay, Egypt. Mediterranean Marine Science. 2005;6(1): 89–98.

[36] Gherardi M, Lepore E, Sciscioli M, Mercurio M, Licciano M, Giangrande A. A study on spermatogenesis of three Mediterranean serpulid species. Italian Journal of Zoology. 2011;78(2): 174–181.

[37] Staub C. A century of research on mammalian male germ cell meiotic differentiation *in vitro*. J Androl. 2001;22(6): 911–926. doi: 10.1002/j.1939-4640.2001.tb03430.x 11700854 10.1002/j.1939-4640.2001.tb03430.x

[38] Hunter D, Anand-Ivell R, Danner S, Ivell R. Models of in vitro spermatogenesis. Spermatogenesis. 2012;2(1): 32–43. doi: 10.4161/spmg.19383 22553488 10.4161/spmg.19383 PMC3341244

[39] Parks JE, Lee DR, Huang S, Kaproth MT. Prospects for spermatogenesis in vitro. Theriogenology. 2003;59(1): 73–86. doi: 10.1016/S0093-691X(02)01275-X 12499019 10.1016/s0093-691x(02)01275-x

[40] Miura T, Yamauchi K, Takahashi H, Nagahama Y. Hormonal induction of all stages of spermatogenesis in vitro in the male Japanese eel (*Anguilla japonica*). Proc Natl Acad Sci U S A. 1991;88(13): 5774–5778. doi: 10.1073/pnas.88.13.5774 2062857 10.1073/pnas.88.13.5774 PMC51960

[41] Sakai N. Transmeiotic differentiation of zebrafish germ cells into functional sperm in culture. Development. 2002;129(14): 3359–3365. 12091306 10.1242/dev.129.14.3359

[42] Sasaki T, Watanabe A, Takayama-Watanabe E, Suzuki M, Abe H, Onitake K. Ordered progress of spermiogenesis to the fertilizable sperm of the medaka fish, *Oryzias latipes*, in cell culture. Development, Growth & Differentiation. 2005;47(2): 87–97. doi: 10.1111/j.1440-169x.2005.00785.x 15771628 10.1111/j.1440-169x.2005.00785.x

[43] Saiki A, Tamura M, Matsumoto M, Katowgi J, Watanabe A, Onitake K. Establishment of in vitro spermatogenesis from spermatocytes in the medaka, *Oryzias latipes*. Dev Growth Differ. 1997;39(3): 337–344. doi: 10.1046/j.1440-169X.1997.t01-2-00009.x 9227900 10.1046/j.1440-169x.1997.t01-2-00009.x

[44] Nagahama Y, Miura T, Kobayashi T. The Onset of Spermatogenesis in Fish Ciba Foundation Symposium 182—Germline Development: John Wiley & Sons, Ltd.; 2007 pp. 255–270. 10.1002/9780470514573.ch14 7835154

[45] Grier HJ. Sperm development in the teleost *Oryzias latipes*. Cell Tissue Res. 1976;168(4): 419–431. doi: 10.1007/bf00215993 1277277 10.1007/BF00215993

[46] Sato T, Katagiri K, Gohbara A, Inoue K, Ogonuki N, Ogura A, et al In vitro production of functional sperm in cultured neonatal mouse testes. Nature. 2011;471(7339): 504–507. doi: 10.1038/nature09850 21430778 10.1038/nature09850

[47] Bertout M. Endocrine regulation of spermatogenesis in *Nereis diversicolor* (Annelida Polychaeta): Experimental study of the control of meiotic differentiation. J Exp Zool. 1983;226(1): 151–161. doi: 10.1002/jez.1402260118 6189962 10.1002/jez.1402260118

[48] Levine H, Jørgensen N, Martino-Andrade A, Mendiola J, Weksler-Derri D, Mindlis I, et al Temporal trends in sperm count: a systematic review and meta-regression analysis. Hum Reprod Update. 2017;1: 1–14. doi: 10.1093/humupd/dmx022 10.1093/humupd/dmx022 PMC6455044 28981654

